# From Cation Order to Disorder: Unlocking Ion Transport Pathways in Li–Zn–Zr–Cl Halospinels

**DOI:** 10.1021/acs.chemmater.6c00461

**Published:** 2026-06-25

**Authors:** Abby M. Cardoza, Tyler B. Case, Christopher L. Rom, Karina Davis, David M. Halat, Annalise E. Maughan

**Affiliations:** † Department of Chemistry, 3557 Colorado School of Mines, Golden, Colorado 80401, United States; ‡ 53405 National Laboratory of the Rockies, Golden, Colorado 80401, United States

## Abstract

Lithium metal chloride halospinels of the general formula Li_2_ *M*Cl_4_ are a promising class of earth-abundant ion conductors for all-solid-state batteries. However, poor room-temperature ionic conductivity has historically limited their use in practical applications. Here, we substitute Zr^4+^ into Li_2_ZnCl_4_ along the series Li_2–2*x*/3_Zn_1–*x*_Zr_2*x*/3_Cl_4_ (*x* = 0, 0.1, 0.3, 0.4, 0.6, 0.9, and 1.0) to understand how cation disorder and vacancy tuning impact ion transport in “normal” halospinels. Aliovalent Zr^4+^ substitution increases ionic conductivity by nearly 5 orders of magnitude, from 1.320(3) × 10^–9^ S cm^–1^ in Li_2_ZnCl_4_ to 6.74(1)× 10^–5^ S cm^–1^ for *x* = 0.6. Average and local structure characterization through synchrotron X-ray diffraction (SXRD) and neutron pair distribution function (nPDF) analysis reveal that Zr^4+^ redistributes the Zn^2+^ and Li^+^ sublattices into previously unoccupied interstitial sites, which form new low-energy hopping pathways that facilitate ion transport. We rationalize the dramatic rearrangement of the cation local structure by considering the coordination preferences of the cations and the potential electrostatic penalties incurred by the higher-valent Zr^4+^ cations. This work delivers an atomistic understanding of substitution-induced cation disorder and ion transport properties in a new family of earth-abundant halospinels.

## Graphical abstract

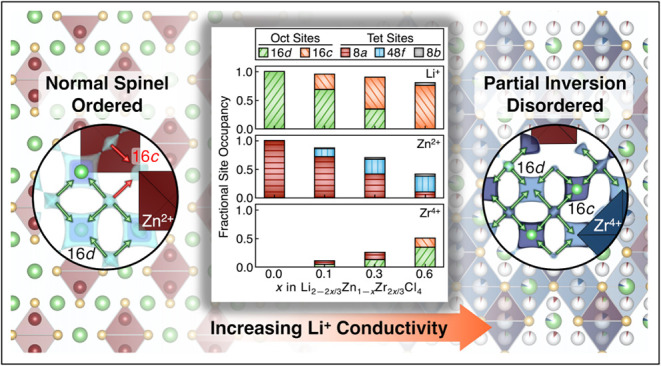

## Introduction

All-solid-state batteries hold the potential to advance energy storage technologies by replacing the flammable liquid electrolyte with a solid-state ion conductor. Solid-state electrolytes (SSEs) may enable the use of Li metal anodes, which are estimated to increase battery energy density by 70%.^1^ Yet, their widespread utilization is limited by the lack of SSEs made from earth-abundant chemistries that simultaneously exhibit room-temperature ionic conductivities comparable with liquid electrolytes (>10^–2^ S cm^–1^) and wide electrochemical stability windows that enable their use with both lithium metal anodes and high-voltage cathodes (0–4 V vs Li/Li^+^).^2,3^

Ternary lithium metal chlorides, such as Li_3_ *M*Cl_6_ and Li_2_ *M*_2/3_Cl_4_ (M = Sc^3+^, Y^3+^, In^3+^), are a family of SSEs offering high oxidative stability (4.3 V vs Li/Li^+^) along with moderate to high ionic conductivities (up to 10^–3^ S cm^–1^).^4–10^ Halospinels are structural derivatives of the ternary metal halide family with the general formula Li_2_ *M*Cl_4_. The cation-deficient Li_2_Sc_2/3_Cl_4_ spinel was recently discovered to exhibit lithium-ion conductivity of 1.5 × 10^–3^ S cm^–1^, which is nearly comparable with liquid electrolytes. The exceptional ionic conductivity of Li_2_Sc_2/3_Cl_4_ arises from the introduction of *M*-site vacancies, which redistribute the lithium ions into interstitial sites and create a large number of low-energy, three-dimensional ion-diffusion pathways.^7^ Halospinels such as Li_2_MgCl_4_ and Li_2_ZnCl_4_ are comprised of substantially more earth-abundant chemistries, but their applicability in all-solid-state batteries has so far been limited by their low room-temperature ionic conductivities (≤10^–7^ S cm^–1^).^11–13^ Recently, aliovalent substitution has been shown to enhance ionic conductivities in halospinels by introducing mobile-ion vacancies and redistributing the cations to facilitate ion transport. For instance, Zr^4+^ substitution in the inverse spinel Li_2_MgCl_4_ results in partial cation occupation onto interstitial sites, which increases lithium-ion conductivity by several orders of magnitude.^13,14^ Similar cation disordering effects were observed in the Li_2–3*x*_MgAl_*x*_Cl_4_ series; the excess Li^+^ vacancies generated by Al^3+^ substitution significantly improved Li^+^ conductivity but also resulted in Mg^2+^ residing on octahedral interstitials (16*c*), which limited lithium transport through those sites.^15^

To date, most studies of earth-abundant halospinels have focused on substitution-induced disorder in inverse spinels. In “inverse” spinels such as Li_2_ *M*Cl_4_ (M = Mg, Ti, Mn, and Cd), Li^+^ cations fully occupy the tetrahedral sites and exhibit split-site occupancy on the octahedral sites with the divalent cations.^12,16–18^ Ion conduction in inverse spinel systems typically proceeds through an oct-tet-oct hopping mechanism between the 8*a* sites and 16*c* interstitial voids.^19^ In contrast, “normal” spinels have *M* ^2+^ cations residing on tetrahedral (8*a*) sites, while Li^+^ ions occupy the octahedral (16*d*) sites (Figure 1). Studies of ion transport in normal halospinels are substantially more scarce than those for inverse spinels. Li_2_ZnCl_4_ is the only lithium chloride spinel to adopt the normal spinel structure and exhibits poor lithium-ion conductivity at room temperature (∼1 × 10^–9^ S cm^–1^).^11,20^ The low mobility of Li^+^ cations arises due to a high activation barrier of the 16*d* to 48*f* interstitial transport pathway (Figure 1). Recent studies of Li_2+*x*_In_*x*_Zn_1–*x*_Cl_4+2*x*_ (0 ≤ *x* ≤ 0.5) and Li_2_Zn_1/3_Zr_1/3_Cl_4_ report increases in room-temperature ionic conductivity up to 9.2 × 10^–4^ S cm^–1^ ^20^ and 5 × 10^–5^ S cm^–1^,^21^ respectively. As halospinels are amenable to extensive cation disorder upon aliovalent substitution, the distinct cation arrangements of normal vs inverse spinels offer new insights into how substitution-induced disorder impacts ion transport.

**Figure 1:**
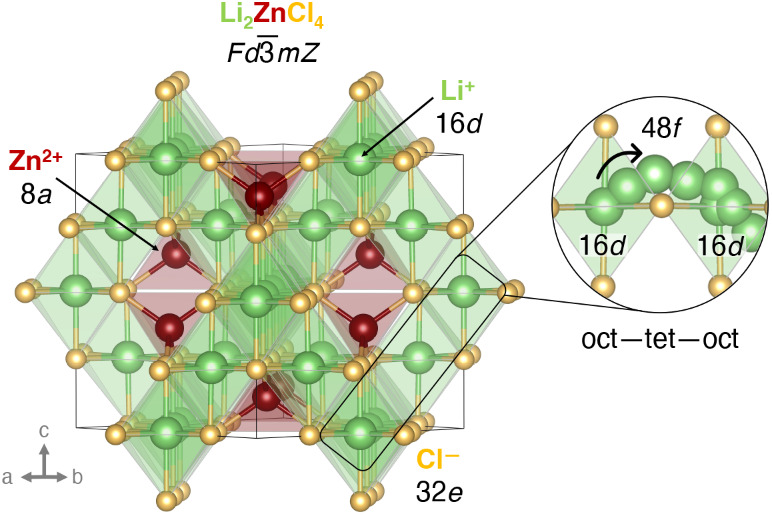
Crystal structure model of the normal halospinel Li_2_ZnCl_4_. Li^+^ resides in octahedral (16*d*) sites, and Zn^2+^ occupies tetrahedral (8*a*) positions. The primary ion conduction pathway in Li_2_ZnCl_4_ is shown in the callout, where Li^+^ travels along an oct-tet-oct pathway between the 16*d* and 48*f* interstitial sites.

In this work, we synthesized the Li_2–2*x*/3_Zn_1–*x*_Zr_2*x*/3_Cl_4_ normal spinel series and correlated the observed rearrangement of the cation local structure with lithium ion transport. We find that the phase-pure spinel structure is maintained up to *x* = 0.6 before reaching a solubility limit. Aliovalent Zr^4+^ substitution enhances lithium ion conductivity by nearly 5 orders of magnitude, from 1.320(3) × 10^–9^ S cm^–1^ in Li_2_ZnCl_4_ to 6.74(1) × 10^–5^ S cm^–1^ for *x* = 0.6. Characterization of the cation local structure through joint synchrotron X-ray diffraction (SXRD)/neutron pair distribution function (nPDF) analysis, paired with Raman and ^6^Li solid-state nuclear magnetic resonance spectroscopy, reveals that Zr^4+^ substitution triggers a rearrangement of the Li^+^, Zn^2+^, and Zr^4+^ cations into interstitial sites. We attribute the substitution-induced spinel inversion and subsequent cation disordering to cation coordination preferences coupled with the strong electrostatic interactions of the highly charged Zr^4+^ species. We further find that the redistribution of the cations creates additional low-energy transport pathways through newly occupied octahedral sites that facilitate transport. This work presents a new compositional family of earth-abundant halospinel solid-state electrolytes and connects the nontrivial changes in the cation local structure to ion transport.

## Results and Discussion

### Synthesis and Crystal Structure of Zr-Substituted Li_2_ZnCl_4_

Members of the Li_2–2*x*/3_Zn_1–*x*_Zr_2*x*/3_Cl_4_ (*x* = 0.0, 0.1, 0.3, 0.4, 0.6, 0.9, and 1.0) spinel series were synthesized by mechanochemical ball milling followed by relatively low-temperature annealing for 10 h and slow cooling at 0.2 °C/min. High-energy planetary ball milling is necessary to encourage initial reactivity and prevent volatilization of the metal chloride precursors, while slow cooling is necessary to avoid the formation of nonspinel impurities (see Figures S1–S3). Unsubstituted Li_2_ZnCl_4_ was annealed at *T* = 200 °C; above *T* = 215 °C, the spinel phase transforms to the olivine polymorph (*Pnma*), which can persist upon cooling.^22^ All Zr-substituted members of the series were annealed at *T* = 150 °C. Similar to the Li–Mg–Zr-Cl spinel analogs, we find that annealing temperatures above *T* = 200 °C promote phase segregation into spinel and Li_2_ZrCl_6_ phases (Figure S4).^13^

Li_2_ZnCl_4_ adopts the cubic, normal spinel structure (space group *Fd*3̅*mZ*: origin 2), as determined by high-resolution synchrotron powder X-ray diffraction (SXRD) collected on the SLAC SSRL BL 2–1 diffractometer (Figure 2a). The structure is characterized by a cubic close-packed arrangement of chloride anions; Zn^2+^ cations fill 1/8 of the tetrahedral voids on the 8*a* Wyckoff site, while Li^+^ cations occupy 1/2 of the octahedral positions on the 16*d* Wyckoff site. The sharp, well-resolved reflections are indicative of high crystallinity and large particle sizes. As shown in Figure 2a, systematic substitution of Zr^4+^ for Zn^2+^ in the Li_2–2*x*/3_Zn_1–*x*_Zr_2*x*/3_Cl_4_ series produces phase-pure cubic spinel for *x* ≤ 0.6. Laboratory powder X-ray diffraction (PXRD) of *x* = 0.9 reveals a mixture of both the spinel phase and the trigonal Li_2_ZrCl_6_ phase (*P*3̅*m*1), which indicates a solubility limit of Zr^4+^ into the spinel structure (Figure S5). For *x* ≤ 0.6, Zr^4+^ substitution results in reduced diffraction intensities and substantial broadening of the characteristic spinel reflections. In particular, we note the systematic disappearance of the 111 and 311 reflections, similar to our prior work in the Li–Mg–Zr–Cl series.^13^ From Pawley refinements of the spinel structure, we observe a linear increase in lattice parameter from *x* = 0.0 to *x* = 0.3, followed by a sharp increase at *x* = 0.4 and *x* = 0.6 (Figure 2b and Figure S6). The sharp increase in the lattice parameters at *x* = 0.4 coincides with the complete disappearance of the 111 and 331 reflections in the diffraction data. The systematic changes in peak breadth and intensities coupled with the non-Vegardian behavior of the lattice parameters above *x* = 0.3 suggest that Zr^4+^ substitution has a nontrivial impact on the structure. A recent study suggested that Zr^4+^ substitution into Li_2_ZnCl_4_ is accompanied by a distortion from cubic spinel symmetry.^20^ However, we do not observe similar peak splitting in the range of 5 ≤ *Q* ≤ 7 Å^–1^ in our diffraction data. Attempts to fit the reported monoclinic structure resulted in extremely poor fits to our SXRD data, which lends support to our assignment of the cubic spinel structure (Figure S16).

**Figure 2:**
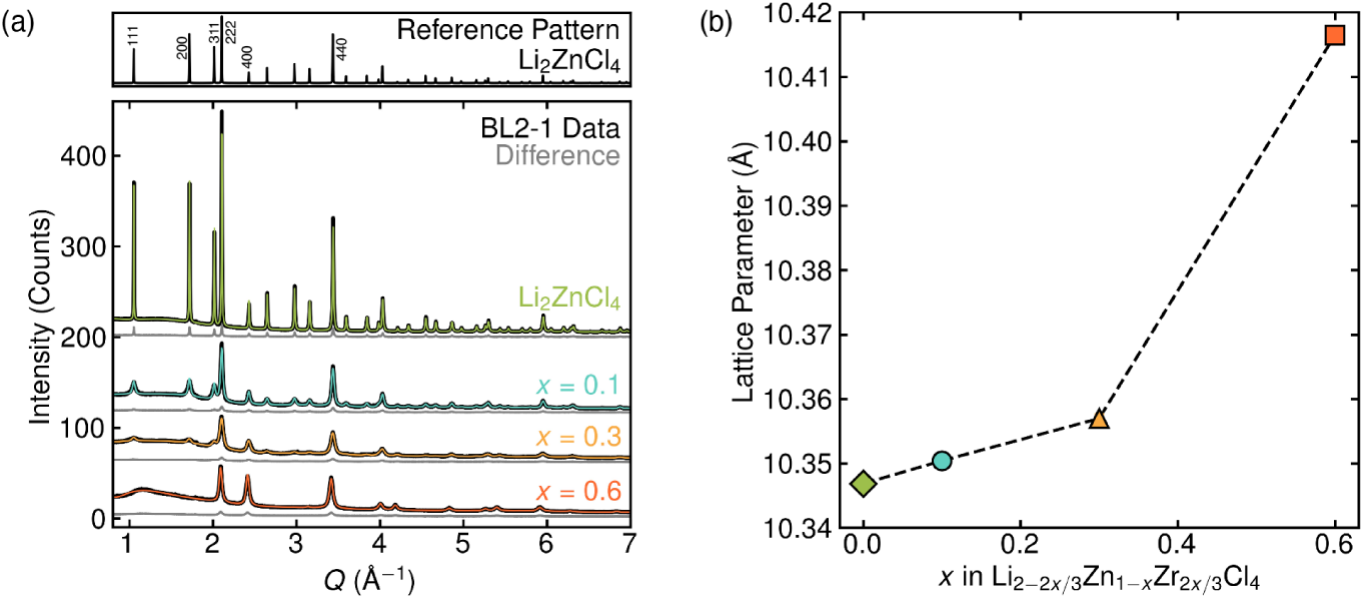
(a) Synchrotron powder X-ray diffraction (SXRD) (black circles) of Li_2–2*x*/3_Zn_1–*x*_Zr_2*x*/3_Cl_4_ at *x* = 0, 0.1, 0.3, and 0.6 collected at SLAC SSRL BL 2–1. Pawley refinements are shown as colored lines. (b) Cubic spinel lattice parameters extracted from Pawley refinements. For all compositions, the error bars are smaller than the symbols. SXRD of the composition at *x* = 0.4 was not collected. However, LeBail fits and lattice parameters extracted from laboratory powder X-ray diffraction (PXRD) across all compositions are shown in Figure S6.

### Lithium-Ion Transport Properties

Lithium-ion transport properties of the Li–Zn–Zr-Cl halospinels were determined through temperature-dependent potentiostatic electrochemical impedance spectroscopy (EIS). Symmetric cells were assembled in a two-electrode geometry with blocking graphite foil electrodes. To extract relevant resistance and capacitance values from the impedance, Nyquist plots were modeled using the equivalent circuit method implemented in PyEIS.^23^ Figure 3 shows Nyquist plots and respective fits for each composition at 30 °C with the real (*Z*_real_) and imaginary (−*Z*_imag_) components of the impedance normalized to the cross-sectional area and thickness of the solid electrolyte pellet to enable comparison between compositions.

**Figure 3:**
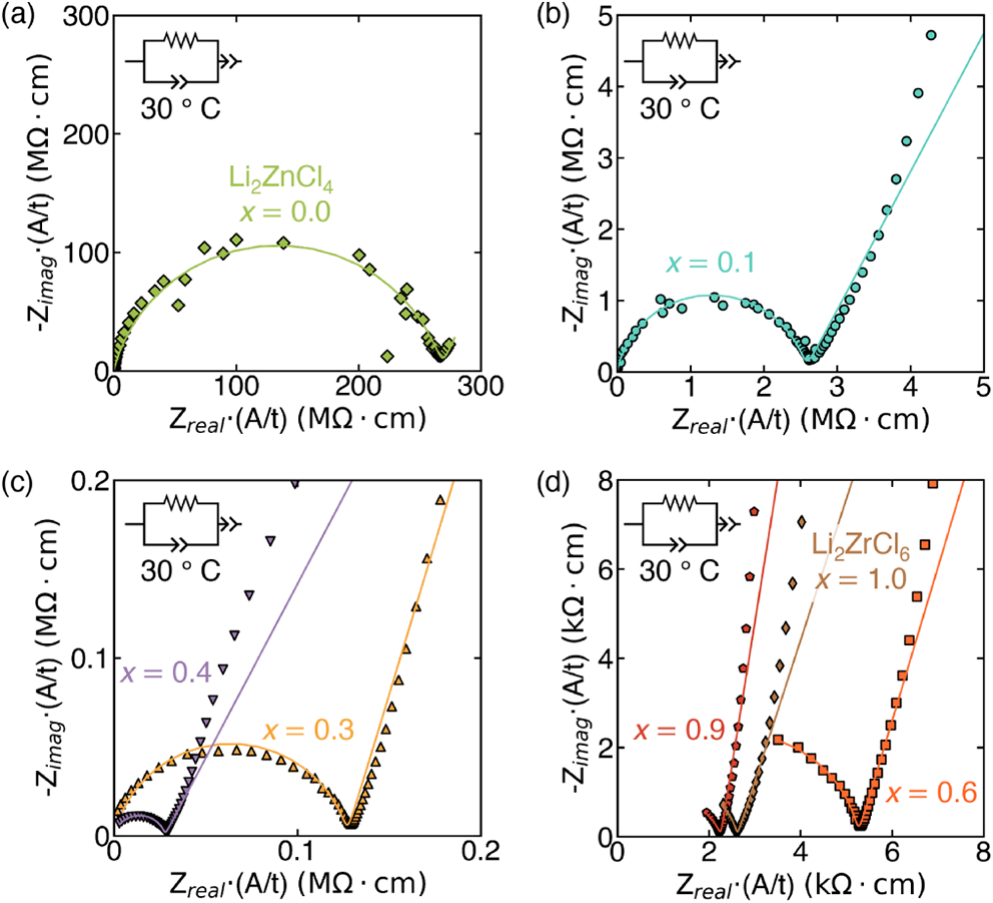
Nyquist plots obtained at *T* = 30 °C for the Li_2–2*x*/3_Zn_1–*x*_Zr_2*x*/3_Cl_4_ series, including (a) Li_2_ZnCl_4_ (*x* = 0), (b) *x* = 0.1, (c) *x* = 0.3, *x* = 0.4, and (d) *x* = 0.6, 0.9, and Li_2_ZrCl_6_ (*x* = 1). The EIS data were modeled with an (*R*_1_ *Q*_1_) + *Q*_2_ equivalent circuit, as shown in the circuit diagram inset. *Z*_real_ and *Z*_imag_ were normalized to the cross-sectional area and thickness of pellets.

The EIS data were quantitatively modeled using an (*R*_1_ *Q*_1_) + *Q*_2_ equivalent circuit; the high-frequency semicircle was modeled with a resistor and constant phase element in parallel (*R*_1_ *Q*_1_), while an additional constant phase element *Q*_2_ in series was included to describe the low-frequency capacitive response. A constant phase element was used as opposed to a capacitor to model nonideality of both the semicircle and capacitive tail resulting from other electrochemical processes occurring at inhomogeneous interfaces between the electrolyte and the blocking electrodes.^24^ Fitting of the (*R*_1_ *Q*_1_) element yields capacitance values of ∼10^–12^ F and ideality parameters ranging from *n* = 0.81–0.92 across all compositions, which together suggest that (*R*_1_ *Q*_1_) is primarily a result of bulk ion transport.^25^ At higher frequencies, the capacitive tail is adequately fit with ideality values of *n* = 0.70–0.90. At lower frequencies, the observed tail changes slope, which may indicate the presence of an additional process occurring at similar frequencies. We suspect that the slight nonideality of the bulk transport is a result of an additional semicircular feature that is overlapping with (*R*_1_ *Q*_1_) and the higher frequencies of the *Q*_2_ element (Figure S15). Although we cannot reliably model this feature due to its subtle appearance, we suspect that it may arise from grain boundary ion transport. In order to avoid overfitting the data, we elected to use the simpler (*R*_1_ *Q*_1_) + *Q*_2_ equivalent circuit. At higher temperatures, compositions yielding the highest ionic conductivities (*x* = 0.9, *x* = 1) lack the semicircle feature and are therefore fit with an *R*_1_+*Q*_2_ equivalent circuit. Fitted parameters and Nyquist plots for all compositions at each temperature can be found in Tables S2–S8 and Figures S8–S14.

The unsubstituted spinel Li_2_ZnCl_4_ exhibits a very low lithium-ion conductivity of σ = 1.320(3) × 10^–9^ S cm^–1^ at *T* = 30 °C (Figure 3a), which is in agreement with recently reported values.^20^ The poor room-temperature conductivity has historically precluded accurate measurement of the lithium-ion transport behavior of Li_2_ZnCl_4_. In the present work, we reduced the overall impedance of the cell by minimizing the thickness of the solid electrolyte pellet (*t* ≤ 1.1 mm) between graphite electrodes and measuring under a constant pressure of 40 MPa. Upon increasing the temperature to *T* = 95 °C, the ionic conductivity of Li_2_ZnCl_4_ increases to σ ∼ 10^–6^ S cm^–1^, which is similar to the early reported values of ∼ 10^–7^ S cm^–1^ at *T* = 100 °C.^11,21^

Aliovalent substitution of Zr^4+^ in the Li_2–2*x*/3_Zn_1–*x*_Zr_2*x*/3_Cl_4_ spinel series exponentially increases the room-temperature Li^+^ conductivity. For *x* = 0.1, incorporation of only 2.3 mol % Zr^4+^ increases the lithium-ion conductivity at 30 °C by 2 orders of magnitude from 1.320(3) × 10^–9^ S cm^–1^ to 1.39(3) × 10^–7^ S cm^–1^, as shown in Figure 3b. Higher substitution fractions in *x* = 0.3 and *x* = 0.6 result in an additional order-of-magnitude increase for each measured composition, yielding σ = 2.79(1) × 10^–6^ S cm^–1^ and σ = 6.74(1) × 10^–5^ S cm^–1^, respectively (Figure 3c,d). The ionic conductivity of the Li_2_ZrCl_6_ end member was found to be σ = 1.355(3) × 10^–4^ S cm^–1^, which is in close agreement with previously reported values.^13,26–28^ Interestingly, the mixed-phase composition at *x* = 0.9 exhibits a slightly higher ionic conductivity (1.570(5) × 10^–4^ S cm^–1^) compared to Li_2_ZrCl_6_. Various studies have shown positive impacts on ion conduction in mixed-phase solid electrolytes.^29,30^ The increase in conductivity observed at *x* = 0.9 may be due to large mobile-ion concentrations at heterogeneous interfaces and high degrees of disorder that promote fast ion transport.

Temperature-dependent EIS measurements reveal that bulk ion transport in the spinel series follows the Arrhenius relationship σ*T* = σ_0_exp­(-*E*_a_/*k*_B_ *T*), as shown in Figure 4. Aliovalent Zr^4+^ substitution results in a substantial and systematic decrease in the activation energy (*E*_a_) from 0.99(21) eV for Li_2_ZnCl_4_ to 0.44(2) eV for *x* = 0.6. The unsubstituted spinel Li_2_ZnCl_4_ exhibits deviations from the Arrhenius relationship, resulting in a poor linear fit and a calculated slope much higher than reported values of 0.85 eV (Figure 4a).^11^ We find temperatures up to 85 °C result in an activation energy of 0.79(13) eV, which is consistent with previous reports. However, above 85 °C, there is a jump to higher conductivities. This same break in Arrhenius behavior has previously been observed by Artal et al., where around 75–85°C, the activation energy in Li_2_ZnCl_4_ changes from 0.89(8) eV to 0.59(1) eV.^20^ This behavior has been attributed to a potential structural order–disorder transition occurring at elevated temperatures. Based on the slight deviation from Arrhenius behavior at, we suspect Li_2_ZnCl_4_ at 85 °C resides at the cusp of this transition point.

**Figure 4:**
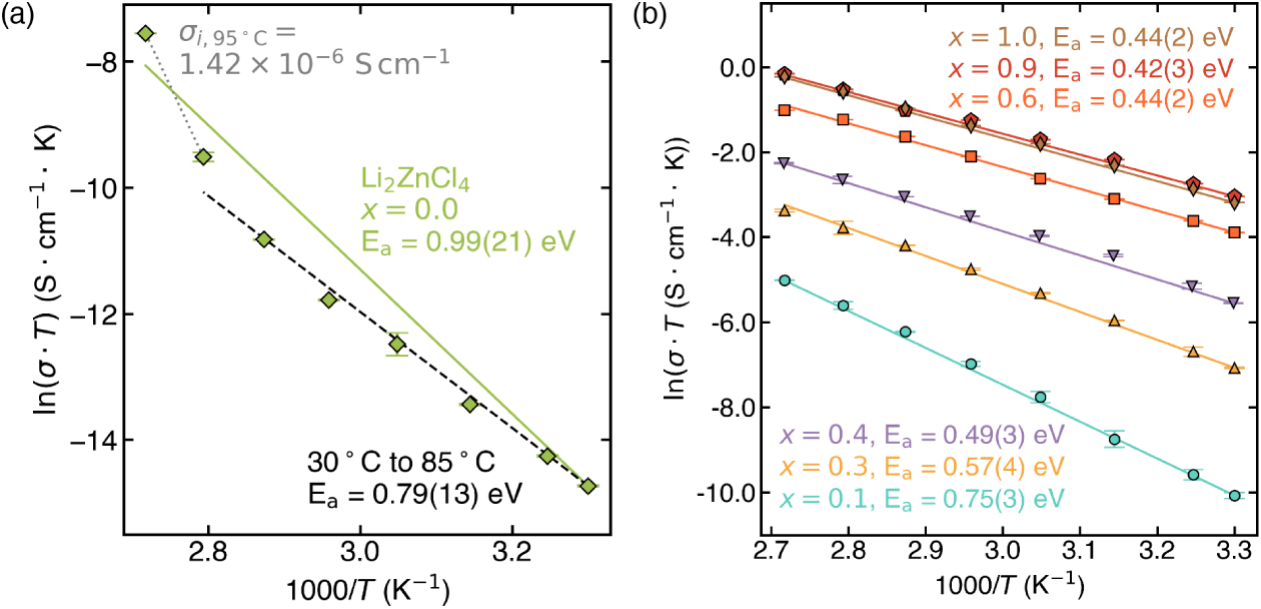
Arrhenius relationships for lithium ion transport for (a) Li_2_ZnCl_4_ and (b) across the Li_2–2*x*/3_Zn_1–*x*_Zr_2*x*/3_Cl_4_ series for *x* = 0.1, 0.3, 0.4, 0.6, 0.9, and 1.0.

Aliovalent Zr^4+^ substitution yields a substantial increase in lithium-ion conductivity across the series. Figure 5 shows trends in room-temperature ionic conductivity (σ), activation energy (*E*_a_), and Arrhenius prefactor (σ_0_) as a function of *x* across Li_2–2*x*/3_Zn_1–*x*_Zr_2*x*/3_Cl_4_. We observe an increase in ionic conductivity with increasing Zr^4+^ substitution, concomitant with a decrease in the activation energy for bulk transport. Following a sharp increase from Li_2_ZnCl_4_ to *x* = 0.1, the Arrhenius prefactor (σ_0_) decreases monotonically between *x* = 0.1 and 0.6. The decrease of both activation barrier and Arrhenius prefactor from *x* = 0.1–0.6 is consistent with Meyer–Neldel compensation behavior (Figure S7).^31^ The changes in both activation barrier and Arrhenius prefactor yield a nearly 5 orders of magnitude increase in ionic conductivity for *x* = 0.6. For Li_2_ZnCl_4_, the nonmonotonic changes in the Arrhenius prefactor may be indicative of changes in ion transport pathways and carrier density arising from Zr^4+^ substitution. Furthermore, as *x* = 0.9 and *x* = 1 are beyond the solubility limit for Zr^4+^ into the spinel structure, deviation from the Meyer–Neldel compensatory behavior is expected due to differences in ionic transport regimes between the cubic spinel and trigonal *P*3̅*m*1 Li_2_ZrCl_6_ structures. The ionic transport parameters are summarized in Table 1. Aliovalent substitution is well-known to influence ion transport by modulating interstitial occupancies, cation disorder, and redistribution of the lithium sublattice.^32^ In order to unravel the mechanism behind the drastic increase in Li^+^ conductivity of these materials, detailed analysis of the local bonding and crystal structure is required.

**Table 1:** Summary of Electrochemical Characterization Results at 30 °C for Ionic Conductivity (*σ*), Activation Energy (*E*_a_), and Arrhenius Prefactor (*σ*_0_) Extracted from (*R*_1_ *Q*_1_) + *Q*_2_ Equivalent Circuit^a^

Composition	*x*	σ_30 °C_ (S/cm)	*E*_a_ (eV)	Prefactor, σ_0_ (S K cm^–1^)
Li_2_ZnCl_4_	0	1.320(3) × 10^–9^	0.79(13)	5.6(17) × 10^6^
Li_1.93_Zn_0.90_Zr_0.07_Cl_4_	0.1	1.39(3) × 10^–7^	0.75(3)	1.20(6) × 10^8^
Li_1.80_Zn_0.70_Zr_0.20_Cl_4_	0.3	2.79(1) × 10^–6^	0.57(4)	2.4(2) × 10^6^
Li_1.73_Zn_0.60_Zr_0.27_Cl_4_	0.4	1.280(4) × 10^–5^	0.49(3)	5.4(5) × 10^5^
Li_1.60_Zn_0.40_Zr_0.40_Cl_4_	0.6	6.74(1) × 10^–5^	0.44(2)	4.6(3) × 10^5^
Li_1.40_Zn_0.10_Zr_0.60_Cl_4_	0.9	1.57(1) × 10^–4^	0.42(3)	5.4(4) × 10^5^
Li_2_ZrCl_6_	1.0	1.36(1) × 10^–4^	0.44(2)	7.5(3) × 10^5^

a Calculated values and errors are based on 3 replicate measurements for each composition.

**Figure 5:**
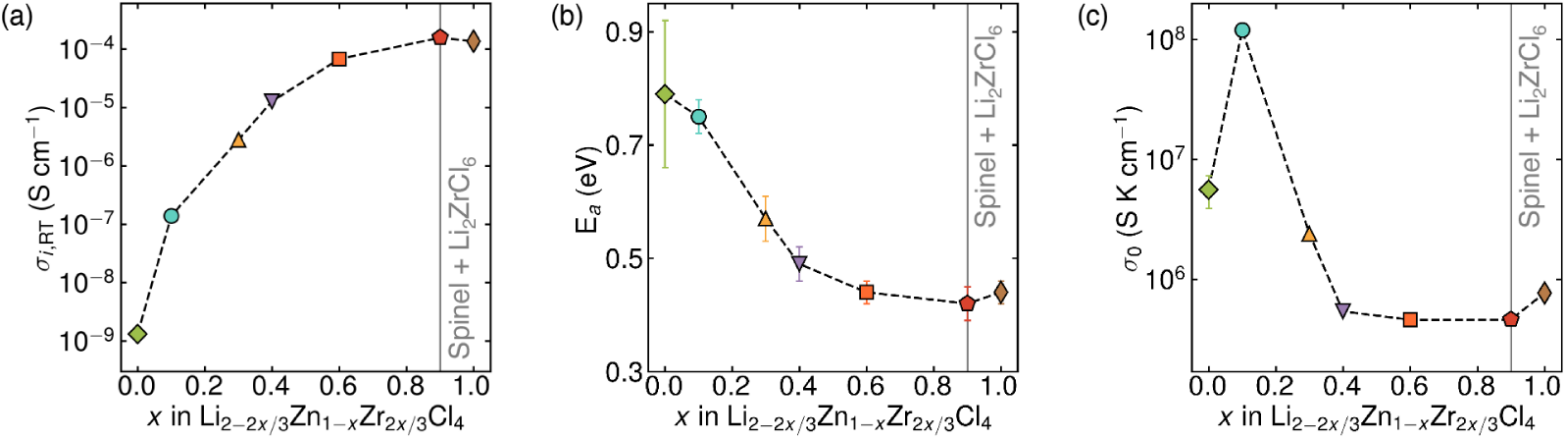
Trends in (a) ionic conductivity (σ), (b) activation energy (E*_a_*), (c) and Arrhenius prefactor (σ_0_) across Li_2–2*x*/3_Zn_1–*x*_Zr_2*x*/3_Cl_4_ at 30 °C.

### Investigation of the Local Structure

#### Joint Synchrotron X-ray Diffraction/Neutron Total Scattering Refinements

To understand how aliovalent Zr^4+^ substitution impacts the cation local structure in the Li_2–2*x*/3_Zn_1–*x*_Zr_2*x*/3_Cl_4_ series, we collected neutron total scattering data suitable for neutron pair distribution function (nPDF) analysis on the NOMAD diffractometer (Spallation Neutron Source, Oak Ridge National Laboratory) and synchrotron X-ray diffraction from BL 2–1 at SLAC SSRL. We performed joint structural refinements with both SXRD data and nPDF analysis to capture both average and local structures with complementary sensitivity to both high-*Z* elements (Zn^2+^, Zr^4+^, Cl^–^) and lithium. Initial Zr^4+^-substituted models were constructed with Zr^4+^ sharing 16*d* octahedral sites with Li^+^ and no cation occupancy on the 16*c*, 48*f*, and 8*b*. However, Rietveld refinements of these models with the SXRD data reveal significant over-modeling of the 200 and 311 reflections and under-modeling of the 222 reflection, which results in excess electron density on all interstitial sites in electron density Fourier difference maps (Figure 6). These observations are consistent with a rearrangement of the local structure upon substitution, in which cations are distributed over interstitial sites with substantial fractional occupancy.

**Figure 6:**
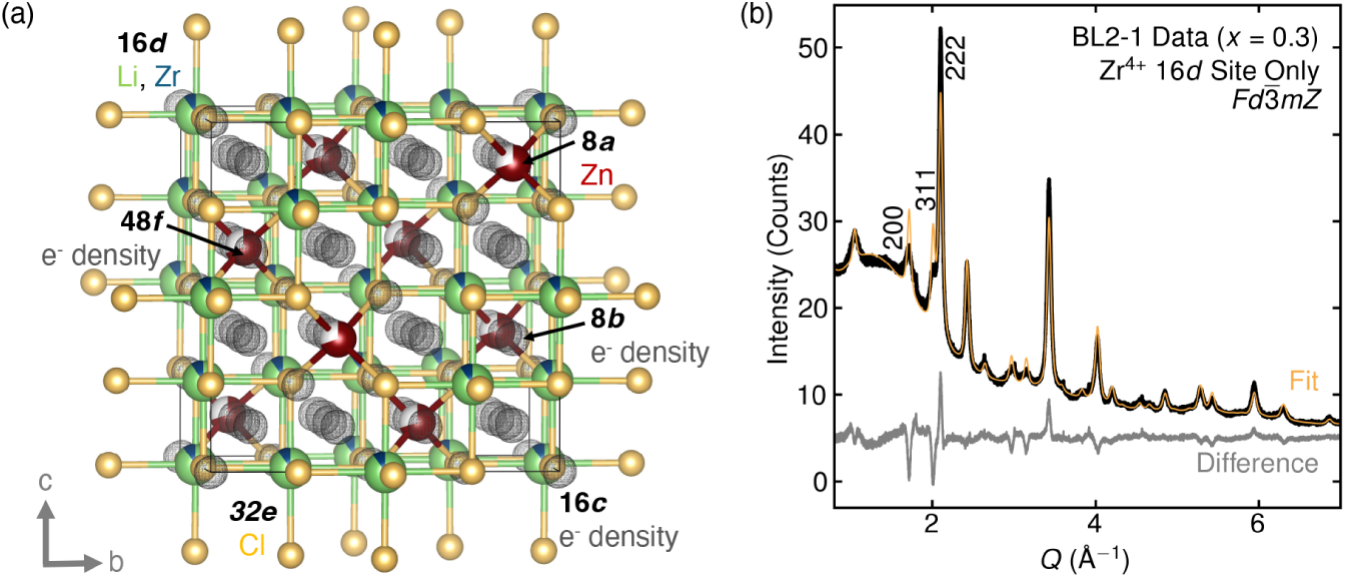
(a) Electron density Fourier difference map of *x* = 0.3 overlaid on the spinel structure showing excess electron density on the 8*a*, 48*f*, and 8*b* sites. (b) SXRD and Rietveld refinement of *x* = 0.3 with Zr^4+^ only on 16*d* sites.

In order to capture the complexity of the cation local coordination environment, we constructed refinements in which Li^+^, Zn^2+^, and Zr^4+^ were permitted to occupy the regular cation sites in the spinel structure (16*d*, 8*a*) as well as the interstitial sites (16*c*, 8*b*, 48*f*) for *x* = 0–0.6. *x* = 0.9 is a mixed phase (spinel and Li_2_ZrCl_6_) and is therefore excluded from these fits. The relative amount of Zr^4+^ was allowed to refine under minimal restrictions, while the total amount of Li^+^ and Zn^2+^ was constrained to the stoichiometry determined by the substitution series (Li_2–2*x*/3_Zn_1–*x*_Zr_2*x*/3_Cl_4_). More information regarding fitting parameters can be found in the Supporting Information. The total concentration of Zr^4+^ across all potential sites refined to values similar to the synthesis stoichiometry (Tables S10–S13), which lends support to the robustness of our modeling approach. Fits from the final refined models to the SXRD and nPDF data for *x* = 0–0.6 are shown in Figure 7.

**Figure 7:**
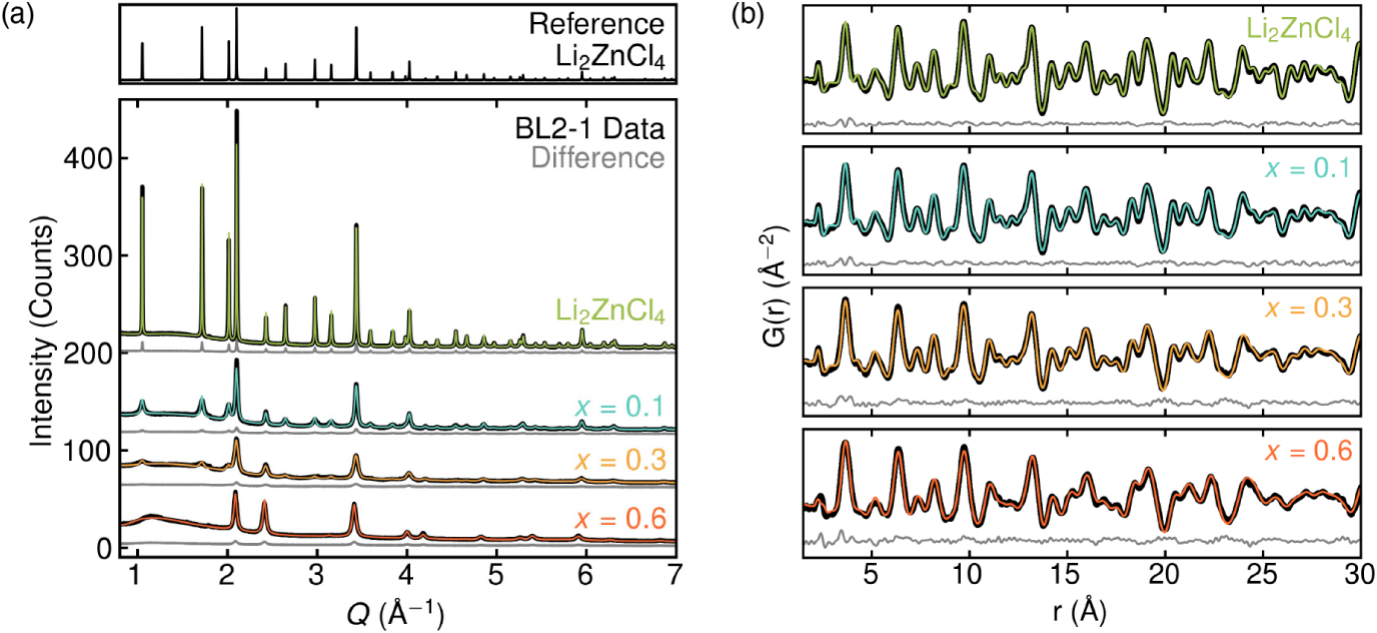
(a) Synchrotron X-ray diffraction (SXRD) and (b) neutron pair distribution function (nPDF) joint refinements across Li_2–2*x*/3_Zn_1–*x*_Zr_2*x*/3_Cl_4_ for *x* = 0.0, 0.1, 0.3, and 0.6. Experimental data are shown as black circles, fits from joint refinements are shown in colored lines, and the difference curves are shown in gray.

The SXRD and nPDF of Li_2_ZnCl_4_ are well modeled by the normal spinel structure, with Zn^2+^ on 8*a* tetrahedral sites, Li^+^ on the 16*d* octahedral sites, and no interstitial occupancy (Figure 7). However, substitution of Zr^4+^ results in redistribution of the cations across interstitial sites. The refined fractional occupancies of each cation across the five possible sites across the series are shown in Figure 8. For *x* = 0.1 and *x* = 0.3, we find that Zr^4+^ cations reside on both octahedral (16*d*) and tetrahedral (8*a*) sites. Zn^2+^ cations reside predominantly on the native 8*a* sites but begin to occupy tetrahedral interstitials (48*f*, 8*b*) upon Zr^4+^ substitution. Lithium ions reside primarily in octahedral 16*d* and 16*c* sites. Interestingly, further increasing the concentration of Zr^4+^ in the *x* = 0.6 composition results in partial spinel inversion, with Zr^4+^ exclusively occupying octahedral sites (16*c*, 16*d*) rather than tetrahedral sites. At this composition, Li^+^ no longer resides in the octahedral 16*d* site and instead occupies the 16*c* and 8*b* sites. Zn^2+^ atoms reside across the tetrahedral 8*a*, 48*f*, and 8*b* sites. The onset of spinel inversion at *x* = 0.6 is also consistent with the nonlinear increase in the lattice parameter at this composition (Figure 2b).

**Figure 8:**
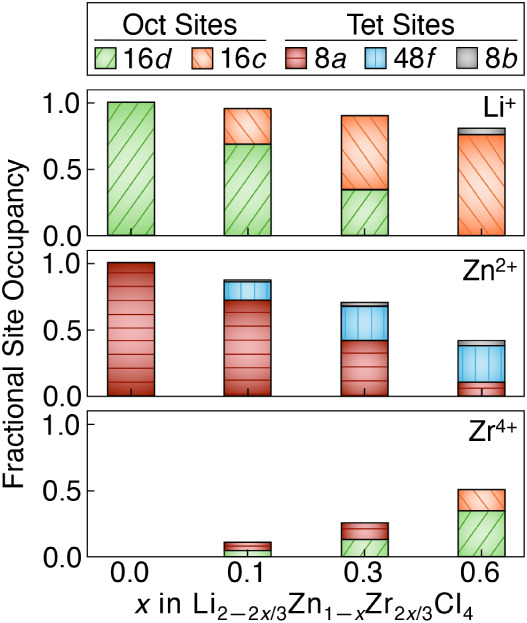
Fractional occupancies of Li^+^, Zn^2+^, and Zr^4+^ in the 16*d*, 16*c*, 8*a*, 8*b*, and 48*f* sites were determined by joint SXRD/nPDF refinements across the Li_2–2*x*/3_Zn_1–*x*_Zr_2*x*/3_Cl_4_ series.

As shown in Figure 7, the distribution of cations across these sites yields excellent fits to both the SXRD and nPDF; in particular, the intensities of the 200, 311, and 222 reflections in the SXRD data are captured by these disordered models. We further refined these models against neutron powder diffraction (NPD) data collected on the POWGEN diffractometer (Spallation Neutron Source, Oak Ridge National Laboratory), as shown in Figure S18. We note that joint refinements with SXRD/nPDF/NPD were not possible due to slight differences in lattice parameters between separate synthesis batches for SXRD/nPDF and NPD. However, we found that the NPD data are well described by the joint-refinement models.

#### Raman Spectroscopy

Raman spectroscopy further illustrates the systematic changes in the Zn^2+^/Zr^4+^ cation coordination environments across the series. The Raman spectra for all members of the series are shown in Figure 9. The Li_2_ZnCl_4_ spinel has five allowed Raman-active modes associated with the ZnCl_4_^2–^ tetrahedra, including the *A*_1*g*_, *E*_*g*_, and 3 *F*_2*g*_ modes.^33,34^ We observe strong characteristic peaks at 291 cm^–1^ (*F*_2*g*_ (1)), 103 cm^–1^ (*E*_*g*_), and 177 cm^–1^ (*F*_2*g*_ (2)) that are in agreement with previous reports.^35,36^ The *F*_2*g*_ (3) mode is reported to occur at 79 cm^–1^; however, our air-free sample holder results in significant background noise at lower wavenumbers and obscures this feature in our data. The Raman spectrum of the Li_2_ZrCl_6_ end member exhibits signatures of the ZrCl_6_^2–^ octahedra, including the *A*_1*g*_ and *F*_2*g*_ modes at 326 cm^–1^ and 165 cm^–1^.^28,37^

**Figure 9:**
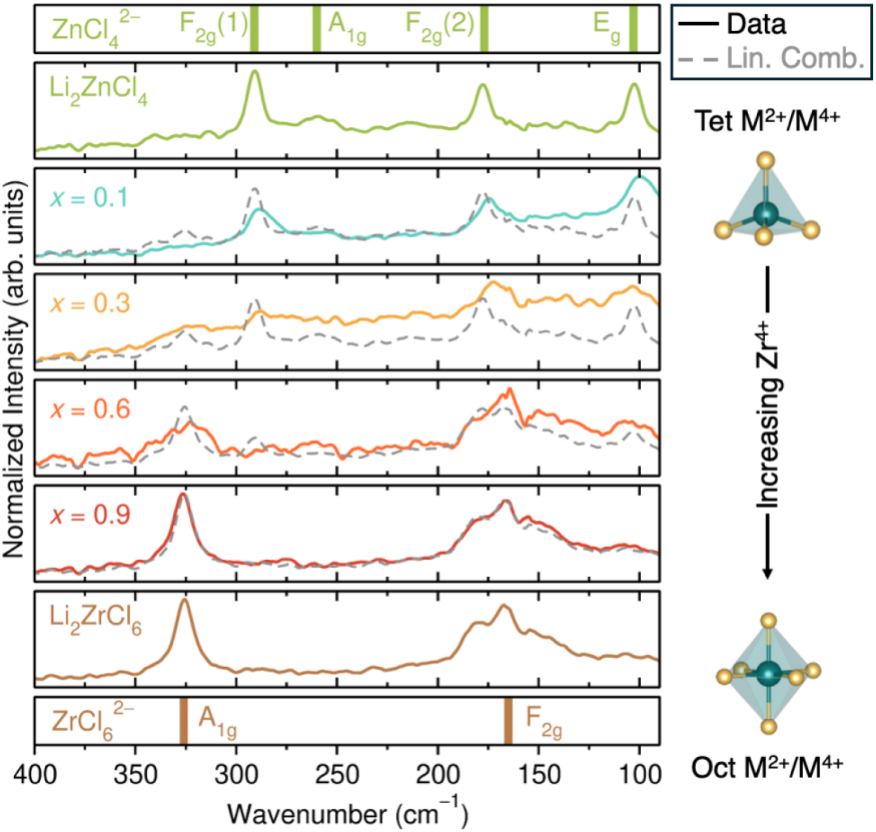
Raman spectra across the Li_2–2*x*/3_Zn_1–*x*_Zr_2*x*/3_Cl_4_ series. Data are shown as colored lines. The ${\mathrm{ZnCl}}_{4}^{2-}$ and ${\mathrm{ZrCl}}_{6}^{2-}$ vibrational modes in Li_2_ZnCl_4_ and Li_2_ZrCl_6_ are shown as green and brown tick marks, respectively. Gray dashed lines represent weighted linear combinations of the Raman spectra of the Li_2_ZnCl_4_ and Li_2_ZrCl_6_ end members.

Substitution of Zr^4+^ for Zn^2+^ is accompanied by shifting and broadening of the spinel vibrational modes, consistent with changes in the local bonding environment that broaden the distribution of vibrational energies.^38^ For *x* = 0.1, the characteristic features of the ZnCl_4_^2–^ tetrahedra in the spinel structure are still present but are broadened and shifted to lower wavenumbers. This observation is consistent with the SXRD data, which suggest that this composition still retains the normal spinel structure, with metal cations residing predominantly on tetrahedral sites. The broadening and shifting of these modes to lower wavenumbers suggest softening of the vibrational landscape upon substitution of the larger Zr^4+^ ions residing on both octahedral and tetrahedral sites (Figure 8). At larger substitution fractions of Zr^4+^, the features in the Raman spectra are further broadened. Features consistent with metal cation occupation on octahedral sites begin to emerge, particularly for *x* = 0.6. For *x* = 0.9, the Raman spectrum resembles that of Li_2_ZrCl_6_, and suggests that Zn^2+^/Zr^4+^ cations reside largely on octahedral sites.

In order to quantify the evolution of the metal cation bonding environment, we modeled the Raman spectra of the intermediate compositions as a weighted linear combination of the Li_2_ZnCl_4_ and Li_2_ZrCl_6_ experimental spectra. The weighting coefficients were determined by an *ad hoc* least-squares analysis and serve as a proxy for the relative fraction of the Zn^2+^ and Zr^4+^ cations in octahedral vs tetrahedral coordination environments. The resulting linear combination spectra are shown as dashed gray lines in Figure 9. As shown in Figure 10, the relative fraction of metal cations on tetrahedral sites decreases concomitantly with an increase in the fraction of cations on the octahedral sites. These trends are closely mirrored by the changes in cation site occupancies determined by structural refinements from both the SXRD and nPDF (Figure 7). These data illustrate that incorporation of higher-valent Zr^4+^ cations is structurally nontrivial and triggers a redistribution of the cations across multiple sites in the structure.

**Figure 10:**
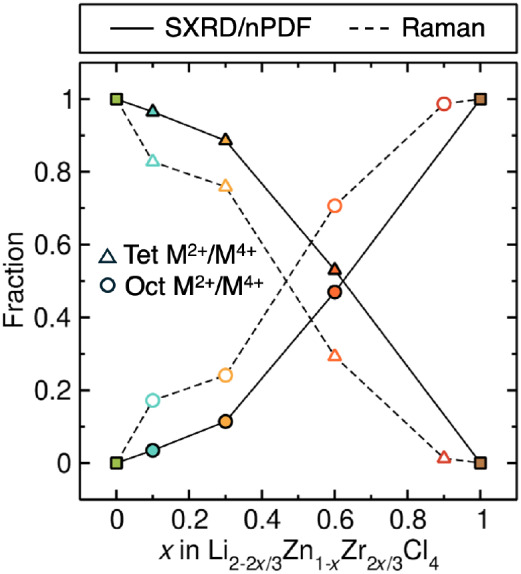
Relative fraction of Zn^2+^/Zr^4+^ residing on octahedral (circles) vs tetrahedral (triangles) sites determined by joint structural refinements from SXRD/nPDF (solid lines) and from linear combination analysis of the Raman data (dashed lines).

#### ^6^Li Solid-State NMR

In order to understand how the lithium coordination environment evolves with Zr^4+^ substitution, we performed ^6^Li solid-state NMR spectroscopy. ^6^Li NMR is sensitive to subtle changes in the local coordination environment of Li^+^, with improved resolution compared with ^7^Li NMR measurements due to smaller dipolar coupling-induced broadening. ^6^Li NMR has previously been used to identify lithium residing in tetrahedral vs octahedral sites in pure and substituted Li_2_MgCl_4_ and Li_2_ZnCl_4_.^39,40^ As shown in Figure 11, we observe a single Li^+^ resonance for each member of the Li_2–2*x*/3_Zn_1–*x*_Zr_2*x*/3_Cl_4_ series, ranging from 0.07 ppm for *x* = 0 to 0.28 ppm for *x* = 0.6. By contrast, two resonances are clearly observed at ∼1.2 ppm and ∼0.3 ppm in the inverse spinel Li_2_MgCl_4_, arising from tetrahedral and octahedral environments, respectively.^40^ Thus, we assign our single low-frequency feature close to ∼0 ppm as octahedral lithium,^39,40^ which is consistent with our analysis of the local structure that suggests Li^+^ predominantly resides on octahedral sites (Figure 8). We do not observe a second signal at ∼1 ppm that would indicate Li^+^ in a tetrahedral environment, as is commonly observed in inverse spinels such as Li_2_MgCl_4_.^12,13^ However, we note that the refined fraction of Li^+^ on the 8*b* site is quite small (<10 atom %), and it may be challenging to distinguish this feature from the noise of the baseline. These observations indicate that lithium predominantly occupies octahedral sites. The shift of the feature to higher frequencies with increasing Zr^4+^ substitution may be due to changes in the second coordination shell that polarizes the chloride sublattice and deshields the lithium ions. We note that the line width of the single Li^+^ peak decreases with increasing Zr^4+^ content, which may be explained by two possible mechanisms. In this study, we observed a multiple-orders-of-magnitude increase in conductivity from *x* = 0.0 to *x* = 0.6. The increased hopping rate may average out homonuclear dipolar coupling and decrease the line width as a result.^41,42^ Alternatively, the substitution of Zr^4+^ introduces vacancies on Li^+^ sites, which reduces the number of Li^+^ neighbors in the second coordination shell of octahedral Li^+^, thus decreasing the homonuclear dipolar coupling experienced by the ^6^Li nuclei.^40^ Overall, the NMR results support our assertion that Li^+^ resides primarily in octahedral sites in the Li–Zn–Zr–Cl normal spinels.

**Figure 11:**
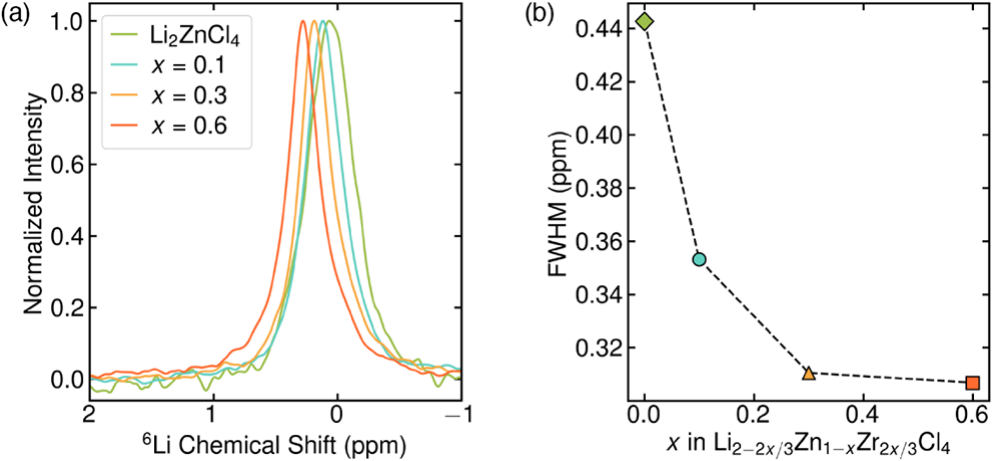
(a) ^6^Li solid-state MAS NMR spectra of Li_2_ZnCl_4_, *x* = 0.1, *x* = 0.3, and *x* = 0.6, acquired at a MAS rate of 10 kHz, and (b) changes in FWHM across the substitution series. Spectra are normalized to equivalent maximum intensity.

#### Bond Valence Sum Analysis

The trends in cation coordination can be rationalized by bond valence sum analysis (Figure 12), which shows that Li^+^ and Zn^2+^ are optimally coordinated in octahedral and tetrahedral sites, respectively. In contrast, neither octahedral nor tetrahedral coordination satisfies the valence of Zr^4+^ cations, consistent with the observation of Zr^4+^ occupying both tetrahedral and octahedral sites. We therefore propose that the evolution of the Zr^4+^ coordination environment from tetrahedral to octahedral sites across the Li–Zn–Zr-Cl series occurs to compensate for electrostatic interactions of the higher-charge Zr^4+^ species. Due to closer average proximity of Zr^4+^ cations at higher concentrations, electrostatic interactions may be screened by a larger number of surrounding halides or vacancies when Zr^4+^ resides in octahedral sites. This may also rationalize the higher degrees of disorder observed across the substitution series. As more Zr^4+^ is substituted into the Li_2_ZnCl_4_ spinel structure, cations disperse into interstitial voids to minimize energetic penalties of multiple higher-valent species in close proximity. An alternative hypothesis may be that cation radius plays a role in dictating cation disorder. This is supported by the redistribution of Zn^2+^ into smaller tetrahedral sites. However, the ionic radius of Zr^4+^ is slightly smaller than Zn^2+^ (*r*_Zr, CN = 4_ = 0.59 Å, *r*_Zn, CN = 4_ = 0.60 Å)^43^ and Zr^4+^ resides in both octahedral and tetrahedral environments, which suggests that electrostatic interactions are the primary driving force for disorder across the series.

**Figure 12:**
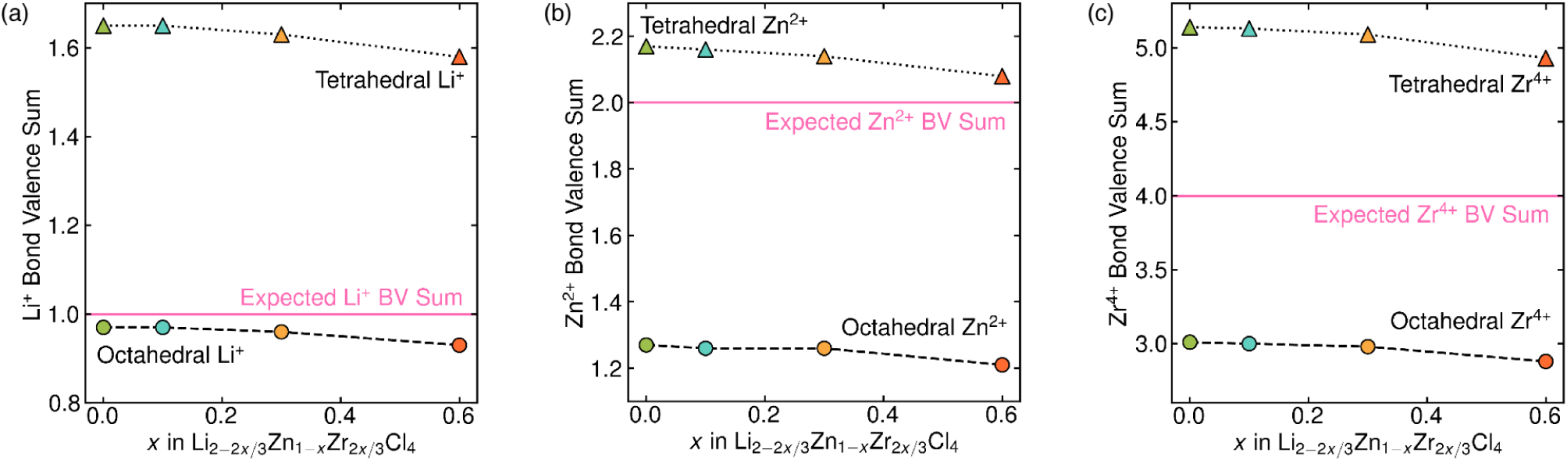
Bond valence sum (BVS) analysis for (a) Li^+^, (b) Zn^2+^, and (c) Zr^4+^. The ideal valence for each cation is shown as a pink line. BVS values were calculated using average octahedral and tetrahedral bond lengths for refined structures. Constants used were *b* = 0.37, *R*_0_ = 1.91 for Li, *R*_0_ = 2.01 for Zn, and *R*_0_ = 2.33 for Zr.^44^ More information regarding calculations and analysis of bond valence sums can be found in the Supporting Information.

### Influence of Cation Local Structure on Ion Transport

To investigate how Zr^4+^ substitution in Li_2_ZnCl_4_ impacts the energetics and availability of possible transport pathways, we utilized bond valence site energy (BVSE) and bond valence path analyzer (BVPA) implemented in softBV.^45,46^ We performed BVSE analysis on both the ordered Li_2_ZnCl_4_ structure (Figure 13a,c) and a representative structure with full cation occupancies for *x* = 0.6 (Figure 13b,d). The representative model for the disordered *x* = 0.6 composition was generated using the *Supercell* program.^47,48^ The cations and vacancies were permuted across the four sites (8*a*, 16*d*, 16*c*, 48*f*) while maintaining the overall stoichiometry. Due to low cation occupancy, Zn^2+^ cations on the 8*b* positions cannot be modeled without a large supercell and were therefore excluded from the permutations; the fractional occupancy of Zn^2+^ cations on the 8*b* site is small (≤10%) and therefore has a negligible impact on the resulting local structure. We extracted migration energies of unique oct-tet hopping pathways to compare differences in ion transport for both *x* = 0 and *x* = 0.6 models.

**Figure 13:**
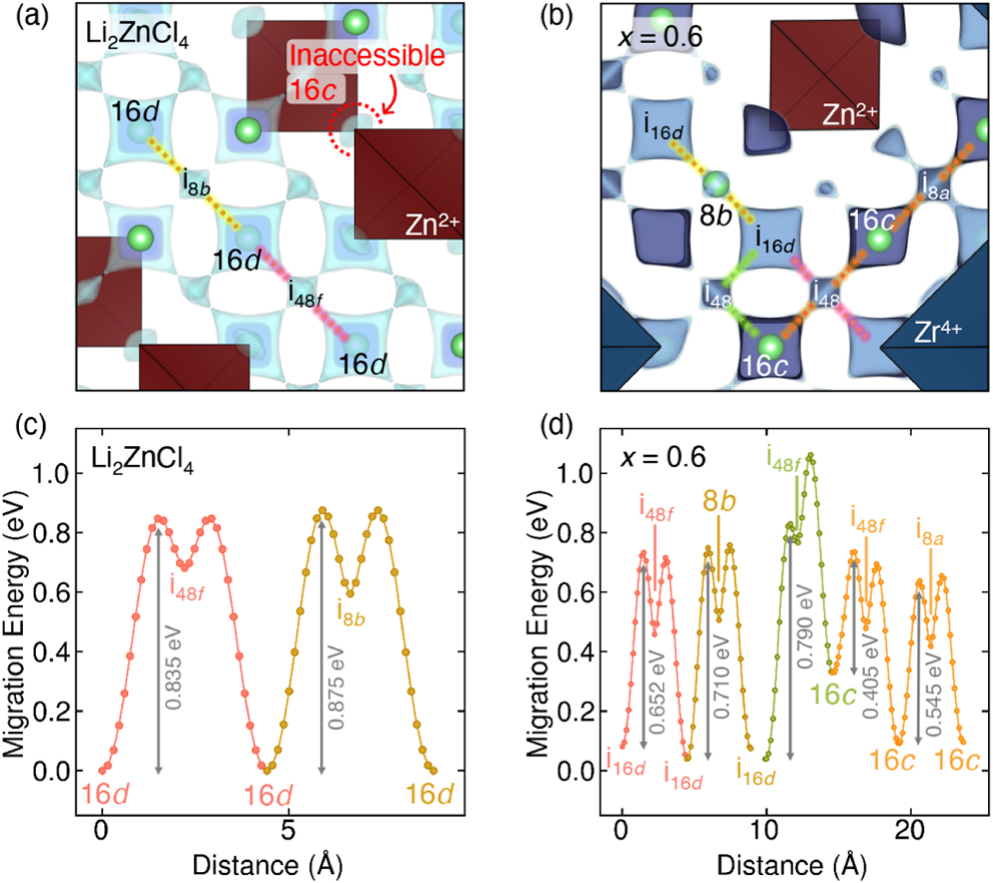
Bond valence site energy (BVSE) landscapes (blue isosurfaces) for (a) Li_2_ZnCl_4_ and (b) *x* = 0.6, highlighting different Li^+^ transport pathways (dotted colored lines). Migration energies for different ion conduction pathways for (c) Li_2_ZnCl_4_ and (d) *x* = 0.6; the colors of the data are coordinated with those in panels (a) and (b).

In ordered spinel structures, mobile ions migrate through an oct-tet-oct hopping pathway (Figure 1). As Li^+^ resides in octahedral sites in normal halospinels (e.g., Li_2_ZnCl_4_), tetrahedral interstitials (48*f*, 8*b*) are the intermediates in the ion conduction pathways. Figure 13a,c shows BVSE analysis of Li_2_ZnCl_4_. In agreement with previous reports, Li^+^ hopping occurs between octahedral 16*d* and either the 48*f* or 8*b* tetrahedral interstitial sites.^11,20^ Both pathways exhibit high migration barriers of 0.835 eV (48*f*) and 0.875 eV (8*b*), consistent with the poor Li^+^ transport observed experimentally (Figure 13b). The slightly higher migration energies associated with 16*d*-8*b* pathways are a result of shorter 8*b* Cl^–^ bond distances (2.196 Å) compared to 48*f* (2.254 Å), which decrease the bottleneck size for Li^+^ ions.^32^ Furthermore, the octahedral 16*c* interstitial sites do not participate in Li^+^ transport pathways due to the close proximity of two tetrahedral Zn^2+^ cations in neighboring tetrahedra that result in high migration barriers (Figure 13a).^11,49^ Similarly, the proximity of Zn^2+^ in the 8*a* sites results in higher site energies for the 48*f* interstitial sites (Figure 13c). In Li_2_ZnCl_4_, each tetrahedral intermediate therefore has only half of its nearest-neighbor sites available for ion hopping. The limited number of possible hopping pathways, coupled with their relatively high migration barriers, provides an atomistic explanation for the low Li^+^ conductivity of Li_2_ZnCl_4_ observed experimentally.

BVSE analysis for *x* = 0.6 reveals distinct differences in available ion transport pathways and energetics due to cation redistribution and partial spinel inversion that promotes faster ion conduction relative to Li_2_ZnCl_4_. To directly compare with Li_2_ZnCl_4_, we visualize the 16*d*-48*f* and 16*d*-8*b* pathways. As shown in Figure 13d, the migration energy for both pathways in *x* = 0.6 decreases to 0.652 eV for 16*d*-48*f* and to 0.710 eV for 16*d*-8*b*. A common metric for explaining ion conduction in disordered spinel structures is the classification of tetrahedral intermediates based on surrounding environments.^49–52^ Tetrahedral sites with all four face-shared octahedral sites occupied by a Li^+^ or a vacancy tend to have lower site energies that decrease the overall migration barrier in an oct-tet hop. In contrast, intermediates that share faces with higher-charged cations (e.g., transition metals) exhibit increased migration energies directly proportional to the number of nearest-neighbor transition metals. In *x* = 0.6, Zr^4+^ occupies both the 16*c* and 16*d* octahedral sites, leaving ∼87% of the octahedral 16*d* sites vacant and available for Li^+^ transport. Additionally, the reduced concentration of Zn^2+^ on the tetrahedral sites creates more accessible octahedral sites for ion hopping compared to Li_2_ZnCl_4_. The decrease in migration energies for the 16*d*-48*f* and 16*d*-8*b* pathways can therefore be attributed to an increase in surrounding mobile octahedral sites that lower tetrahedral intermediate site energies. It is interesting to note that both octahedral (16*c*, 16*d*) sites emerge as local minima in the BVSE calculations (Figure 13d), yet we do not observe Li^+^ occupation on the 16*d* site from structural refinements. Attempts to refine Li^+^ on the 16*d* site resulted in worse fits to the SXRD and nPDF data (Figure S19). BVSE calculations indicate that the estimated migration barriers surrounding the 16*d* site are higher than those of 16*c*, which may provide a qualitative explanation for why we do not observe Li^+^ occupancy on the 16*d* site experimentally. We recognize that the specific local configuration of cations has critical importance for the energetics and accessibility of these pathways.^10,52,53^ However, an in-depth analysis of cation short-range order is beyond the scope of the present work.

Figure 13b illustrates that the introduction of Zr^4+^ and Zn^2+^/Li^+^ vacancies expands the network of transport pathways beyond the 16*d*, 8*b*, and 48*f* sites to include the 16*c* and 8*a* sites that are inaccessible in unsubstituted Li_2_ZnCl_4_. The expanded network of ion transport pathways, paired with decreased migration energies, rationalizes the substantial increase in ionic conductivity from 1.320(3) × 10^–9^ S cm^–1^ for Li_2_ZnCl_4_ to 6.74(1) × 10^–5^ S cm^–1^ for *x* = 0.6 observed experimentally. The observation that partial spinel inversion in *x* = 0.6 is beneficial for transport pathways is further supported by the generally higher ionic conductivities observed in the analogous inverse spinel Li_2–*z*_Mg_1–3*z*/2_Zr_*z*_Cl_4_ series.^13^

## Conclusions

Disorder is ubiquitous in fast-ion conductors. Understanding the crystal-chemical origins of atomic site disorder and how it modifies ion hopping pathways is essential to designing solid-state ion conductors with desirable transport properties. In this study, we investigate the impact of aliovalent substitution on cation disorder and Li^+^ transport in the normal spinel series Li_2–2*x*/3_Zn_1–*x*_Zr_2*x*/3_Cl_4_. Zr^4+^ substitution significantly increases lithium-ion conductivity from ∼1 × 10^–9^ S cm^–1^ for Li_2_ZnCl_4_ up to ∼1 × 10^–4^ S cm^–1^ for *x* = 0.6. We find that Zr^4+^ substitution is accompanied by a significant rearrangement of the cation local structure, in which the cations exhibit partial occupancies on the interstitial sites in the spinel structure. Li^+^ and Zn^2+^ ions reside primarily on octahedral and tetrahedral sites, respectively. In contrast, Zr^4+^ occupies both octahedral and tetrahedral sites at low concentrations of Zr^4+^ (*x* = 0.1, 0.3) but resides entirely on octahedral sites at higher concentrations (*x* = 0.6). We attribute the evolution of the cation local structure and partial spinel inversion to the preferred coordination environments of Li^+^ and Zn^2+^ coupled with the electrostatic penalties associated with higher-charged Zr^4+^ species occupying tetrahedral sites. Comparison of the migration energies of ion hopping pathways between Li_2_ZnCl_4_ and *x* = 0.6 indicates that substitution-induced rearrangement of the cation local structure creates additional, energetically accessible hopping pathways that can facilitate ion transport. Specifically, redistributing Li^+^ ions and vacancies across the octahedral sites enables transport into the 16*c* octahedral interstitial, which is inaccessible in Li_2_ZnCl_4_ due to the electrostatic interactions arising from the close proximity of Zn^2+^ neighbors. Further, distributing Zn^2+^ across multiple tetrahedral sites with partial occupancies opens additional tetrahedral interstitial sites for transport. Taken together, this work delivers a new family of “normal” halospinel solid-state electrolytes and further connects the evolution of the cation local structure with an atomistic understanding of newly formed transport pathways that facilitate macroscopic ionic conductivity.

## Experimental Procedures

### Materials Synthesis

Due to the moisture-sensitive nature of these materials, all manipulations were carried out in an argon-filled glovebox, and all characterization techniques were performed in air-free environments to avoid air exposure and decomposition of the products. Samples were synthesized using a mechanochemical approach coupled with subsequent annealing at low temperatures (150–200 °C). Stoichiometric mixtures (1.5 g scale) of LiCl (Sigma-Aldrich ≥99.98% trace metals basis), ZnCl_2_ (Thermo Scientific, anhydrous, 99.95%), and ZrCl_4_ (Sigma-Aldrich ≥99.9% trace metals basis) were hand-ground in an agate mortar and pestle and then loaded into a 50 mL MSE PRO Y-Stabilized Zirconia Milling (YSZ) vacuum jar containing 142 zirconia milling media (5 mm diameter). The precursor mixtures were milled in an MSE PMV1–0.4L planetary ball mill for 50 cycles of 10 min at 870 rpm followed by a 2 min rest period (10 h total milling time). ∼0.150 g aliquots of the ball-milled precursors were pressed uniaxially into 6 mm diameter pellets at 544 MPa and held at constant pressure for 5 min. Pellets were then sealed in 10 mm diameter fused quartz ampules under vacuum and annealed in a Thermo Scientific Thermolyne FB1415M Muffle Furnace. Li_2_ZnCl_4_ samples were heated to 200 °C at a ramp rate of 5 °C/min. Due to phase separation between the spinel structure and Li_2_ZrCl_6_, substituted materials were instead annealed at 150 °C with a ramp rate of 5 °C/min (Figure S4). All samples were held at the desired temperature for a total of 10 h before cooling to room temperature at a rate of 0.2 °C/min.

### Powder X-ray Diffraction

Laboratory powder X-ray diffraction (PXRD) measurements were collected on a Bruker D2 Phaser benchtop diffractometer with a CuK_α_ source (λ = 1.54 Å). Samples were prepared on a Si zero-diffraction slide and encased in a poly­(methyl methacrylate) (PMMA) airtight Bruker knife-edge dome to prevent air exposure. Synchrotron powder X-ray diffraction (SXRD) measurements were obtained through the SLAC National Laboratory mail-in program for beamline 2–1 at the Stanford Synchrotron Radiation Lightsource (SSRL) at λ = 0.729 Å.^54^ Sample powders were packed into 0.5 mm ID quartz capillaries and flame-sealed under vacuum. The sealed capillaries were nested into 0.7 mm ID polyimide capillaries and sealed with modeling clay. SXRD instrument profiles were extracted from refinement of the NIST 660c LaB6 standard. Le Bail, Pawley, and Rietveld refinements for both PXRD and SXRD were performed using TOPAS v6.^55^

### Neutron Diffraction and Total Scattering

Time-of-flight (TOF) neutron total scattering was collected on beamline BL-1B (NOMAD) at the Spallation Neutron Source, Oak Ridge National Laboratory (SNS ORNL).^56^ Samples were loaded into 3 mm diameter quartz capillaries and sealed using 2-part epoxy. Measurements were collected until a cumulative power (pCharge) of 4 C was reached, corresponding to an estimated measurement time of 50 min. The neutron pair distribution function (nPDF) was calculated through Fourier transformation of total scattering data using a *Q*_max_ of 25 Å. *Q*_damp_ = 0.050(1) Å^–1^ and *Q*_broad_ = 0.028(4) Å^–1^ values used in sample refinements were determined from refinement of the NIST Si 640e standard in TOPAS v6.

Neutron diffraction data were collected from BL-11A (POWGEN) at the Spallation Neutron Source, Oak Ridge National Laboratory (SNS ORNL).^57,58^ Samples were prepared for POWGEN in 6 mm-diameter vanadium cans. Measurements were taken at a constant wavelength of λ = 0.8 Å (Frame 1) for a *d*-spacing coverage of 0.1–8 Å.

### Raman Spectroscopy

Raman spectra were collected using a 1064 nm Thermo Scientific Nicolet iS50 FTIR spectrometer with an FT-Raman module. Samples were prepared by spreading powder on a dual-concavity microscope slide and then sealing the wells with polyimide tape to prevent air exposure. Slides were loaded into the spectrometer with the taped side of the slide facing away from the light source. Measurements for all samples were collected at a resolution of 2 cm^–1^, an aperture of 37, and a range of 0–5391 cm^–1^, with a total of 50 replicate scans at each composition. A dark scan under identical conditions was collected and used for background subtraction. Details about laser intensities can be found in Table S15.

### AC Electrochemical Impedance Spectroscopy

AC potentiostatic electrochemical impedance spectroscopy (EIS) was conducted using a Gamry Interface 1010E Potentiostat. Samples were prepared by pressing 6 mm diameter pellets uniaxially at 544 MPa, yielding theoretical densities of ∼90% (Table S1). Pellets were sandwiched between 6 mm diameter graphite electrodes and loaded into custom air-free polyether ether ketone (PEEK) cells in parallel-plate capacitor geometry with 6 mm diameter stainless steel rods. A custom pressure jig was used to apply a constant pressure of 40 MPa during the measurements, as measured by an in-line load cell. EIS measurements were collected with an applied bias of 20 mV. The frequency was swept logarithmically from 2 × 10^6^ – 0.2 Hz at 10 measurements per decade. Temperature-dependent measurements were collected at 30 °C and from 35 to 95 °C in 10 °C increments in a Quincy Lab convection oven. Samples were equilibrated at each temperature for a total of 3 h.

### ^6^Li Solid-State NMR

^6^Li solid-state NMR experiments were performed with a Bruker 4 mm HXY probe using a Bruker 9.4 T (400 MHz) NMR spectrometer operating at a Larmor frequency of 58.89 MHz at room temperature. Spectra were acquired using a π/2 one-pulse experiment at a magic-angle spinning (MAS) rate of 10 kHz, with a pulse length of 2.5 μs at a power level of 26 W for all samples. The power level and pulse length were initially optimized on an external solid LiCl reference, referenced to 0.0 ppm. Recycle delays of 60 s were employed for all tested Li_2–2*x*/3_Zn_1–*x*_Zr_2*x*/3_Cl_4_ samples.

### Supercell Generation

Representative models were generated using the *Supercell* program.^47,48^ Due to the relatively low fractional site occupancies of cations across the substitution series, minimum supercell sizes of 1 × 1 × 3 for *x* = 0.1, 0.3, and 1 × 1 × 2 for *x* = 0.6 are necessary to adequately model all cation disorder. However, the expanded unit cells yield a configurational space exceeding the threshold limit of the software (10^15^ configurations), rendering the construction of supercells infeasible without multiple permutations. Exclusion of Zn^2+^ occupancy in the 8*b* site enables a 1 × 1 × 1 supercell generation for the composition at *x* = 0.6, while less Zr^4+^-rich compositions still require larger unit cells. For that reason, only the Zr^4+^ substituted member at *x* = 0.6 is considered for supercell generation. To generate the representative model, the disordered structure of *x* = 0.6 was converted to a *P*1 space group and used as the input for the command-line software. Permutations were run under the *Supercell* commands “–c yes”, to ensure charges are balanced and stoichiometry is maintained and “–n l1” to output the configuration with the lowest Coulombic energy. Under these conditions, cations and vacancies are permuted across the 16*d*, 16*c*, 48*f*, and 8*a* sites to deliver the resulting representative structure.

### BVSE Calculations

BVSE analysis for the ordered Li_2_ZnCl_4_ and representative *x* = 0.6 structures was performed using a bond valence path analyzer (BVPA) implemented in softBV.^45,46^ To avoid potential bias in migration energy comparisons, screening factors in both structures are fixed to 0.703906. This approach employs a simplified static framework to construct percolating pathways from the available mobile ion and vacancy sites. Crystallographic positions composed of nonmobile species are excluded from the pathway analysis, regardless of fractional occupancy. In *x* = 0.6, trace amounts of nonmobile ions reside across all octahedral and tetrahedral positions. Therefore, representative models of the experimentally determined disordered structures are required for the BVSE analysis generation.

## Supplementary Material



## Notes

Results of different synthetic parameters, details for sample preparation for electrochemical impedance spectroscopy measurements, additional Nyquist plots, fitting parameters determined from equivalent circuit modeling of electrochemical impedance spectroscopy data, additional structural refinements and refined parameters, and neutron powder diffraction data. Raw data is available in the Maughan-Lab/Li-Zn-Zr-Cl_Halospinels Github repository (PDF)
