# Supplementary material for: From Cation Order to Disorder: Unlocking Ion Transport Pathways in Li–Zn–Zr–Cl Halospinels
Source: Chem Mater. 2026 Jun 25;38(13):6467–78. doi: 10.1021/acs.chemmater.6c00461 (PMC13374042; doi:10.1021/acs.chemmater.6c00461)
Supplement: Supplementary file 1 [file cm6c00461_si_001.pdf]

Supporting Information for: "From Cation Order to Disorder: Unlocking Ion Transport Pathways in Li-Zn-Zr-Cl Halospinels"

Abby M. Cardoza,† Tyler B. Case,† Christopher L. Rom,‡ Karina Davis,† David M. Halat,†,‡ and Annalise E. Maughan*,†,‡

†*Department of Chemistry, Colorado School of Mines, Golden, Colorado 80401, United States*

‡*National Laboratory of the Rockies, Golden, Colorado 80401, United States*

E-mail: amaughan@mines.edu

Contents

Synthesis Studies 4

Figure S1: Long-Term Annealing of LiCl and $ZnCl_2$ Precursors 4

Figure S2: Synthesis of Li_2ZnCl_4 with Mechanochemical Ball Milling 5

Figure S3: Cooling Rate Adjustments After Ball Mill + Annealing Procedure 6

Figure S4: Heating Profiles of Li-Zn-Zr-Cl Materials 7

Figure S5: Before and After Annealing Across Substitution Series 8

Figure S6: Benchtop XRD LeBail Fits and Lattice Parameters 9

Electrochemical Impedance Spectroscopy 10

Table S1: Dimensions and Measurements of EIS Sample Pellets 10

Figure S7: Meyer-Neldel Plot 11

Figure S8, Table S2: Nyquist Plots and Fitted Parameters for Li_2ZnCl_4 12

Figure S9, Table S3: Nyquist Plots and Fitted Parameters for $x = 0.1$ 13

Figure S10, Table S4: Nyquist Plots and Fitted Parameters for $x = 0.3$ 14

Figure S11, Table S5: Nyquist Plots and Fitted Parameters for $x = 0.4$ 15

Figure S12, Table S6: Nyquist Plots and Fitted Parameters for $x = 0.6$ 16

Figure S13, Table S7: Nyquist Plots and Fitted Parameters for $x = 0.9$ 17

Figure S14, Table S8: Nyquist Plots and Fitted Parameters for Li_2ZrCl_6 18

Figure S15: Fitting EIS Data with an $(R_1Q_1) + (R_2Q_2) + Q_3$ Equivalent Circuit 19

Structural Refinements 20

Figure S16: Monoclinic $P2_1/c$ Fit of $Li_{2-2x/3}Zn_{1-x}Zr_{2x/3}Cl_4$ at $x = 0.3$ 20

Figure S17: Vesta Models for $Li_{2-2x/3}Zn_{1-x}Zr_{2x/3}Cl_4$ (x = 0.0, 0.1, 0.3, 0.6) 21

Table S9: Joint Refinement Parameters 22

Table S10: Li_2ZnCl_4 Refined Parameters 24

Table S11: $Li_{1.92}Zn_{0.88}Zr_{0.08}Cl_4$ (x = 0.12) Refined Parameters 24

Table S12: $Li_{1.81}Zn_{0.71}Zr_{0.19}Cl_4$ (x = 0.29) Refined Parameters 25

Table S13: $Li_{1.63}Zn_{0.43}Zr_{0.38}Cl_4$ ($x = 0.57$) Refined Parameters 25

Figure S18, Table S14: POWGEN Neutron Powder Diffraction 26

Figure S19: $x = 0.6$ Joint Refinement Goodness of Fit by 16d Li^+ Concentration 27

Figure S20: $x = 0.3$ SXRD R_{wp} Heat Maps 28

Raman Spectroscopy 29

Table S15: Measurement Details for Raman Spectroscopy 29

Bond Valence Sums 30

Table S16: Bond Valence Sum Calculation Information 30

Synthesis Studies

Long-Term Annealing of LiCl and $ZnCl_2$ Precursors

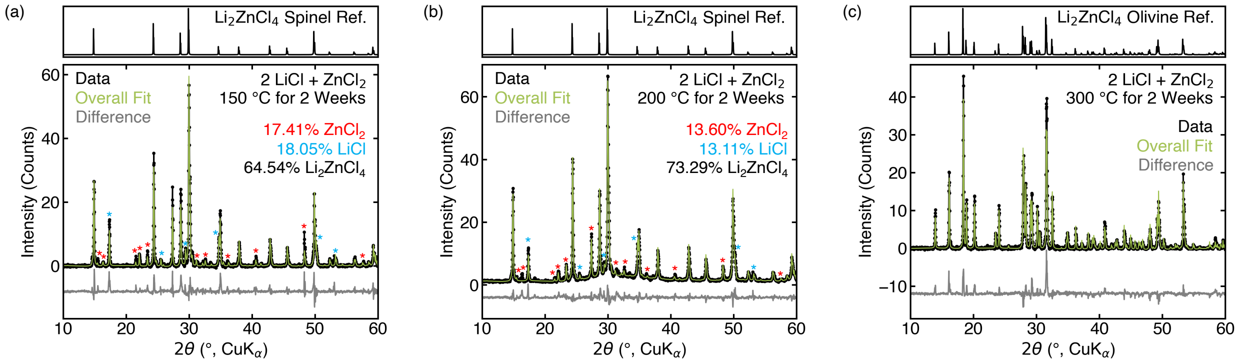

Figure S1: Powder X-ray diffraction (PXRD) Rietveld analysis of Li_2ZnCl_4 synthesis by annealing precursors at (a) 150 °C, (b) 200 °C, and (c) 300 °C for 2 weeks. LiCl and $ZnCl_2$ impurities are marked by blue and red asterisks, respectively

Early studies show the phase pure spinel Li_2ZnCl_4 can be obtained by heating a stoichiometric ratio of precursors at temperatures below the transition point (215 °C) for multiple weeks.[1] To test this, 0.150 g samples were pressed into 6 mm diameter pellets, sealed in quartz ampules and placed in furnaces set at 150, 200, and 300 °C for 2 weeks. Rietveld refinements of laboratory powder X-ray diffraction (PXRD) is shown in Figure S1. At 300 °C, the olivine phase of Li_2ZnCl_4 is formed. At temperatures below the transition point, the spinel phase is obtained along with significant LiCl and $ZnCl_2$ precursor impurities. While both samples annealed at relatively low temperatures demonstrate 2 weeks is not a sufficient duration of time to reach spinel purity, annealing at 200 °C results in less impurities than the sample at 150 °C. For that reason, all synthesized samples of Li_2ZnCl_4 were annealed at 200 °C.

Synthesis of Li_2ZnCl_4 with Mechanochemical Ball Milling

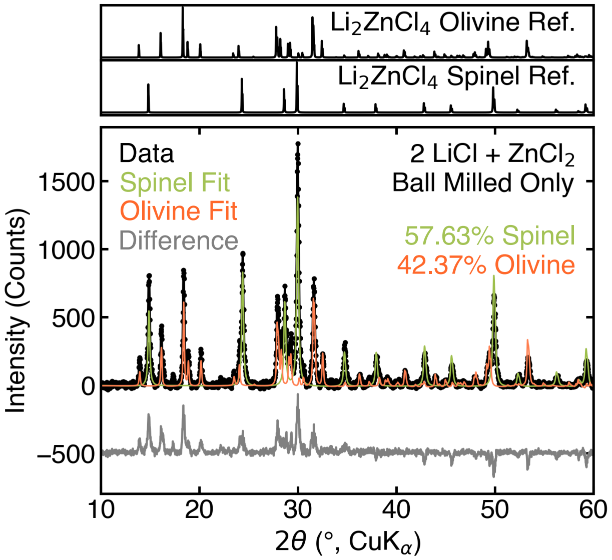

Figure S2: Powder X-ray diffraction (PXRD) Rietveld analysis of a mixed phase Li_2ZnCl_4 sample synthesized with only mechanochemical ball milling. The fit for the spinel phase (Fd$\bar{3}$mZ) is shown in green while the olivine phase (*Pnma*) is depicted in orange.

In addition to long term annealing procedures, the Li_2ZnCl_4 spinel has also been synthesized through mechanochemical ball milling.[2] Precursors LiCl and $ZnCl_2$ were ball milled at 870 rpm for 50 iterations of 10 minutes milling and 2 minutes rest. Powder X-ray diffraction of the ball milled sample reveals a nearly equal mixture between the olivine (*Pnma*) and spinel ($Fd\bar{3}mZ$) phase. The mixed phase composition may be a result of aggressive ball milling parameters. However, lower intensity ball milling procedures result in a large amount of unreacted precursors.

Cooling Rate Adjustments After Ball Mill + Annealing Procedure

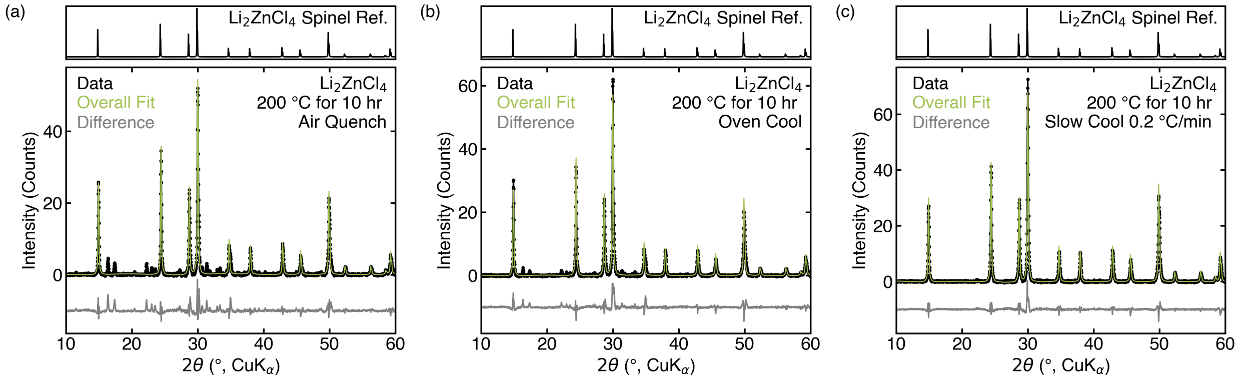

Figure S3: Powder X-ray diffraction LeBail fits for samples of Li_2ZnCl_4 ball milled (BM) and annealed (HT) at 200 °C for 10 hours with different cooling profiles of (a) air quench, (b) furnace cooling, and (c) slow cooling at a rate of 0.2 °C/min.

Samples of Li_2ZnCl_4 were synthesized through ball milling and subsequent annealing at 200 °C for 10 hours. Figure S3 shows powder X-ray diffraction data collected on 3 samples of Li_2ZnCl_4 that are cooled from 200 °C at different rates. Air quenching, the fastest of the cooling rates tested, results in stronger impurity peaks than slower cooling methods. Impurities in the furnace-cooled sample remain present, but are reduced in intensity. It should be noted, there is no definitive rate at which the furnace cools, but based on various time stamps, we predict a rate of about 1 °C per minute for the first hour before slowing down to a rate of about 0.5 °C per minute. When the cooling rate stays at a consistent 0.2 °C per minute, no impurity peaks are observed. Attempts to identify impurity peaks were unsuccessful. However, we believe they may be attributed to an intermediate phase or partial formation of the olivine phase. Since the olivine phase is metastable, rapid cooling or quenching is a common technique to obtain the high temperature polymorph. Slow cooling circumvents kinetic trapping of the olivine phase and enables formation of the spinel.

Heating Profiles of Li-Zn-Zr-Cl Materials

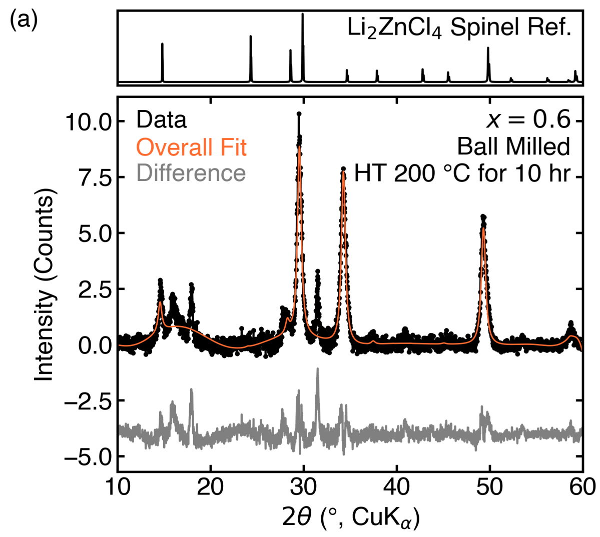

Figure S4: LeBail analysis of powder X-Ray diffraction of ball milled $Li_{2-2x/3}Zn_{1-x}Zr_{2x/3}Cl_4$ (x = 0.6) after 10 hours of annealing at (a) 200 °C and (b) 150 °C.

Figure S4 shows laboratory powder X-ray diffraction data for two different annealing profiles of the sample $Li_{2-2x/3}Zn_{1-x}Zr_{2x/3}Cl_4$ at $x = 0.6$. At 200 °C the material begins to undergo phase separation between the spinel structure ($Fd\bar{3}mZ$) and Li_2ZrCl_6 ($P\bar{3}m1$), presumably due to the volatility of the $ZrCl_4$ precursor. This observation is consistent with the Li-Mg-Zr-Cl analogs.[3] Annealing at 150 °C produces single-phase spinel.

Before and After Annealing Across Substitution Series

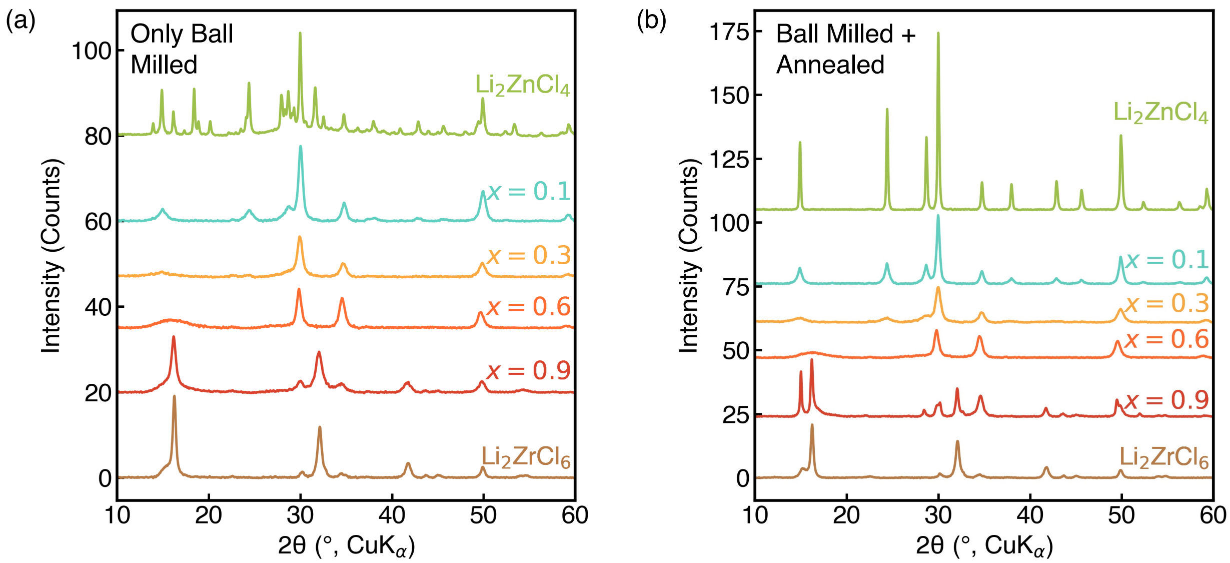

Figure S5: Laboratory powder X-ray diffraction patterns of (a) $Li_{2-2x/3}Zn_{1-x}Zr_{2x/3}Cl_4$ (x = 0.0, 0.1, 0.3, 0.6, 0.9, and 1.0) from only ball milling and (b) after annealing at 150 °C

.

Figure S5 shows compositions across the substitution series before and after annealing of the ball milled precursors. Many compositions only exhibit sharper peaks and more distinguishable features in the diffraction pattern due to an increase in crystallinity following annealing. However for Li_2ZnCl_4 and at x = 0.9, there are significant changes in the diffraction patterns before and after annealing. In Li_2ZnCl_4, the peaks associated with the olivine phase disappear as annealing provides the necessary thermodynamic control to achieve spinel purity. Before annealing, x = 0.9 appears to resemble the trigonal $P\bar{3}m1$ structure of Li_2ZrCl_6. After annealing, the diffraction pattern of x = 0.9 reveals a mixture between the spinel phase and Li_2ZrCl_6.

Benchtop XRD LeBail Fits and Lattice Parameters

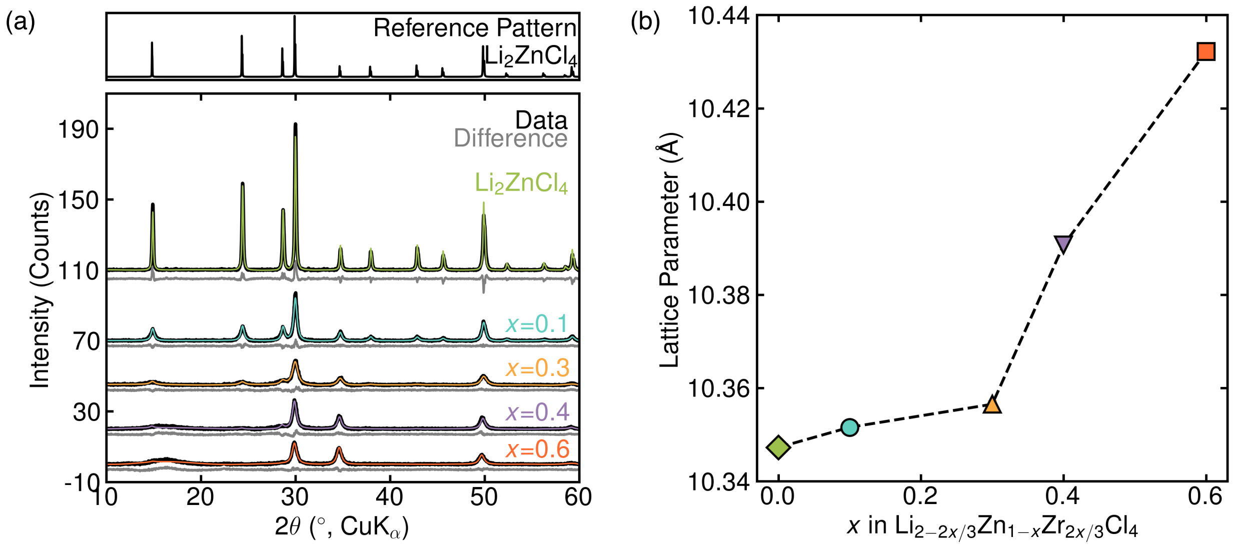

Figure S6: (a) Laboratory powder X-ray diffraction LeBail fits and (b) lattice parameters $Li_{2-2x/3}Zn_{1-x}Zr_{2x/3}Cl_4$ ($x = 0.0$, 0.1, 0.3, 0.4, and 0.6) after ball milling and subsequent annealing.

Electrochemical Impedance Spectroscopy

Dimensions and Measurements of EIS Sample Pellets

Table S1: Dimensions and calculated densities for 6 mm (O.D) diameter pellets pressed at different compositions for electrochemical impedance spectroscopy. Areas (±0.001cm), thicknesses (±0.001 cm), and masses (±0.0001g) of the pellets were measured and used to calculate the pellet densities.

Composition	x	Area (cm^2)	Thickness (cm)	Mass (g)	Density (g/cm^3)	% Theor. Density
Li_2ZnCl_4	0.00	0.2826	0.1065	0.0720	2.41	90.9
$Li_{1.92}Zn_{0.88}Zr_{0.08}Cl_4$	0.12	0.2826	0.1208	0.0812	2.38	90.5
$Li_{1.81}Zn_{0.71}Zr_{0.19}Cl_4$	0.29	0.2826	0.0967	0.0640	2.37	90.8
$Li_{1.73}Zn_{0.60}Zr_{0.27}Cl_4$	0.40	0.2826	0.1761	0.1092	2.19	87.1
$Li_{1.63}Zn_{0.43}Zr_{0.38}Cl_4$	0.57	0.2826	0.1285	0.0815	2.24	88.2
$Li_{1.40}Zn_{0.10}Zr_{0.60}Cl_4$	0.90	0.2826	0.0839	0.0538	2.27	88.0
Li_2ZrCl_6	1.00	0.2826	0.1284	0.0847	2.35	89.2

Meyer-Neldel Plot

Figure S7 illustrates the Meyer-Neldel enthalpy-entropy compensatory relationship across cubic spinel phase pure compositions across the substitution series. Li_2ZrCl_6 and the composition at $x =$ 0.9 exhibit an increase in thein the Arrhenius prefactor due to having a mixture of phases. As the Arrhenius prefactor partly depends on the dimensionality and geometry of transport pathways, the lower Arrhenius prefactor in Li_2ZnCl_4 may suggest an introduction of different transport regimes compared to Zr^{4+}-substituted materials.[4]

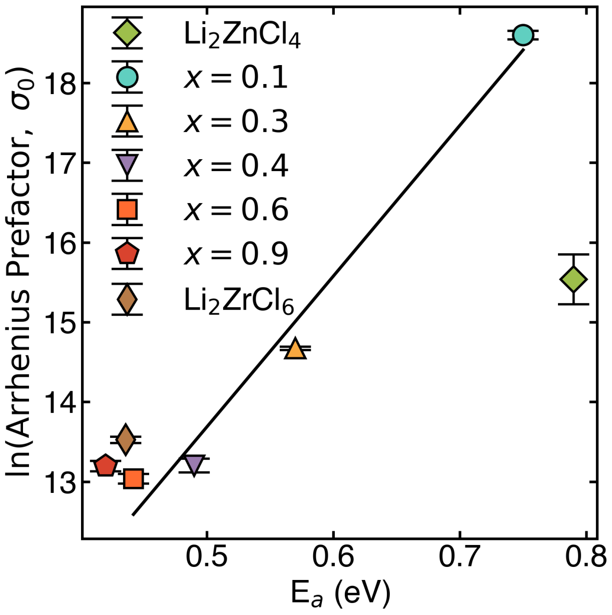

Figure S7: Arrhenius prefactor (σ_0) vs. activation energy (E_a) across $Li_{2-2x/3}Zn_{1-x}Zr_{2x/3}Cl_4$.

Nyquist Plots and Fitted Parameters for Li_2ZnCl_4

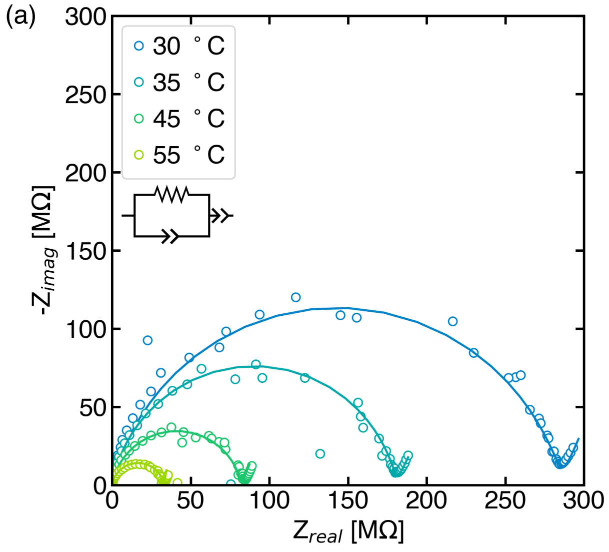

Figure S8: Temperature-dependent Nyquist plots of Li_2ZnCl_4 from (a) 30 °C to 55 °C and (b) 65 °C to 95 °C fit with an $(R_1Q_1) + Q_2$ equivalent circuit.

Table S2: Fitted parameters for Li_2ZnCl_4 of temperature-dependent electrochemical impedance spectroscopy to an $(R_1Q_1)+Q_2$ equivalent circuit, where R_1 is the bulk resistance, Q is a constant phase element, n is the ideality parameter, C is the capacitance, and σ is ionic conductivity.

T (°C)	R_1 (Ω)	σ (S cm^{-1})	n_1	Q_1(S/s^n)	C_1 (F)	τ_1 (Hz)	n_2	Q_2(S/s^n)
30	2.85E+08	1.32E-09	0.85	1.02E-11	3.74E-12	1.49E+02	0.76	2.74E-08
35	1.80E+08	2.09E-09	0.89	1.01E-11	4.64E-12	1.90E+02	0.73	4.45E-08
45	8.20E+07	4.60E-09	0.89	1.17E-11	4.98E-12	3.90E+02	0.75	5.93E-08
55	3.30E+07	1.14E-08	0.90	1.80E-11	7.91E-12	6.10E+02	0.89	8.50E-08
65	1.67E+07	2.26E-08	0.88	1.86E-11	6.4E-12	1.49E+03	0.76	2.28E-07
75	6.59E+06	5.72E-08	0.89	1.51E-11	4.84E-12	4.99E+03	0.70	6.92E-07
85	1.84E+06	2.05E-07	0.89	2.68E-11	7.86E-12	1.10E+04	0.60	2.70E-06
95	2.65E+05	1.42E-06	0.93	1.60E-11	6.32E-12	9.50E+04	0.60	2.00E-05

Nyquist Plots and Fitted Parameters for x = 0.1

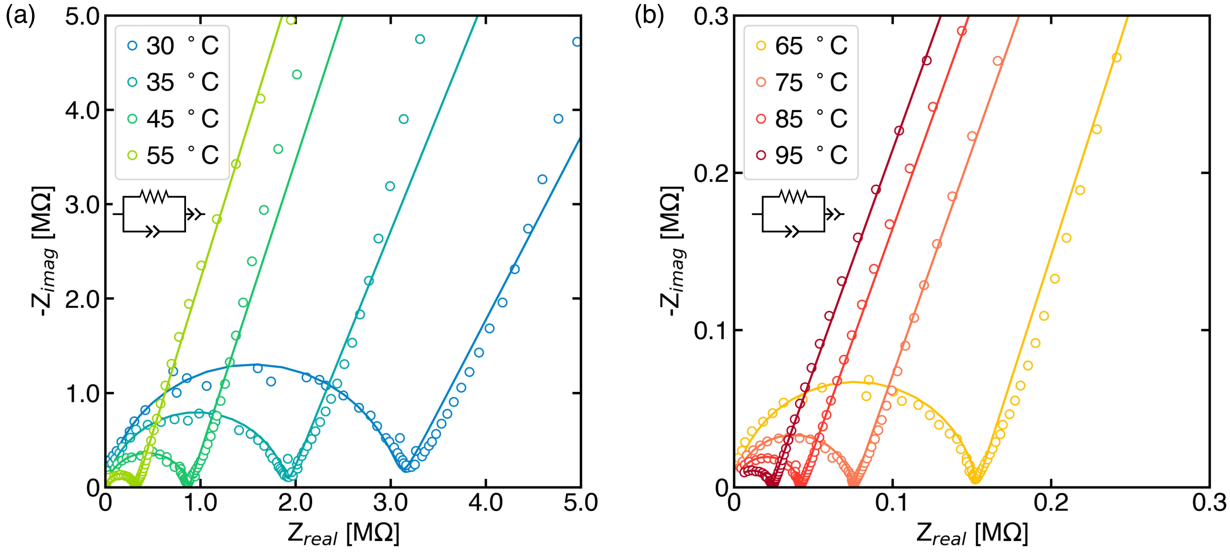

Figure S9: Temperature-dependent Nyquist plots of $Li_{2-2x/3}Zn_{1-x}Zr_{2x/3}Cl_4$ (x = 0.1) from (a) 30 °C to 55 °C and (b) 65 °C to 95 °C fit with an (R_1Q_1) + Q_2 equivalent circuit.

Table S3: Fitted parameters for $Li_{2-2x/3}Zn_{1-x}Zr_{2x/3}Cl_4$ (x = 0.1) of temperature-dependent electrochemical impedance spectroscopy to an $(R_1Q_1) + Q_2$ equivalent circuit, where R_1 is the bulk resistance, Q is a constant phase element, n is the ideality parameter, C is the capacitance, and σ is ionic conductivity.

T (°C)	R_1 (Ω)	σ (S cm^{-1})	n_1	Q_1(S/s^n)	C_1 (F)	τ_1 (Hz)	n_2	Q_2(S/s^n)
30	3.09E+06	1.38E-07	0.89	1.84E-11	5.34E-12	9.65E+03	0.70	6.49E-08
35	1.91E+06	2.23E-07	0.88	2.51E-11	6.42E-12	1.30E+04	0.76	7.30E-08
45	8.81E+05	4.85E-07	0.87	2.37E-11	4.99E-12	3.62E+04	0.80	9.22E-08
55	3.21E+05	1.33E-06	0.92	1.73E-11	6.22E-12	7.98E+04	0.81	1.37E-07
65	1.53E+05	2.79E-06	0.91	1.82E-11	5.51E-12	1.89E+05	0.80	2.10E-07
75	7.51E+04	5.69E-06	0.92	1.91E-11	5.91E-12	3.59E+05	0.79	3.30E-07
85	4.16E+04	1.03E-05	0.95	1.30E-11	6.06E-12	6.32E+05	0.78	4.47E-07
95	2.36E+04	1.81E-05	0.93	1.86E-11	5.82E-12	1.16E+06	0.78	5.73E-07

Nyquist Plots and Fitted Parameters for *x* = 0.3

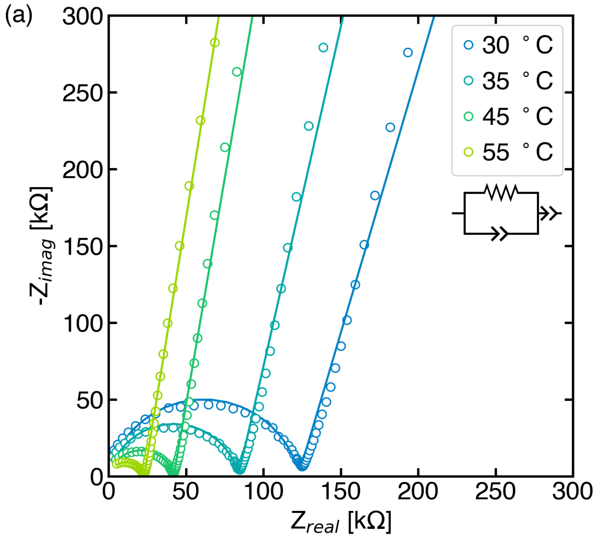

Figure S10: Temperature-dependent Nyquist plots of $Li_{2-2x/3}Zn_{1-x}Zr_{2x/3}Cl_4$ (x = 0.3) from (a) 30 °C to 55 °C and (b) 65 °C to 95 °C fit with an $(R_1Q_1) + Q_2$ equivalent circuit.

Table S4: Fitted parameters for $Li_{2-2x/3}Zn_{1-x}Zr_{2x/3}Cl_4$ (x = 0.3) of temperature-dependent electrochemical impedance spectroscopy to an $(R_1Q_1) + Q_2$ equivalent circuit, where R_1 is the bulk resistance, Q is a constant phase element, n is the ideality parameter, C is the capacitance, and σ is ionic conductivity.

T (°C)	R_1 (Ω)	σ (S cm^{-1})	n_1	Q_1(S/s^n)	C_1 (F)	τ_1 (Hz)	n_2	Q_2(S/s^n)
30	1.23E+05	2.79E-06	0.87	4.65E-11	7.61E-12	1.70E+05	0.82	1.21E-07
35	8.39E+04	4.08E-06	0.87	5.05E-11	7.62E-12	2.49E+05	0.86	1.03E-07
45	4.21E+04	8.13E-06	0.86	6.13E-11	7.61E-12	4.97E+05	0.89	9.51E-08
55	2.29E+04	1.49E-05	0.86	6.91E-11	7.77E-12	8.94E+05	0.90	1.05E-07
65	1.35E+04	2.54E-05	0.85	8.01E-11	7.74E-12	1.53E+06	0.89	1.26E-07
75	7.91E+03	4.32E-05	0.86	7.80E-11	7.97E-12	2.52E+06	0.87	1.80E-07
85	5.31E+03	6.44E-05	0.91	2.65E-11	6.05E-12	4.96E+06	0.83	3.12E-07
95	3.64E+03	9.40E-05	0.86	6.80E-11	5.75E-12	7.60E+06	0.83	3.87E-07

Nyquist Plots and Fitted Parameters for x = 0.4

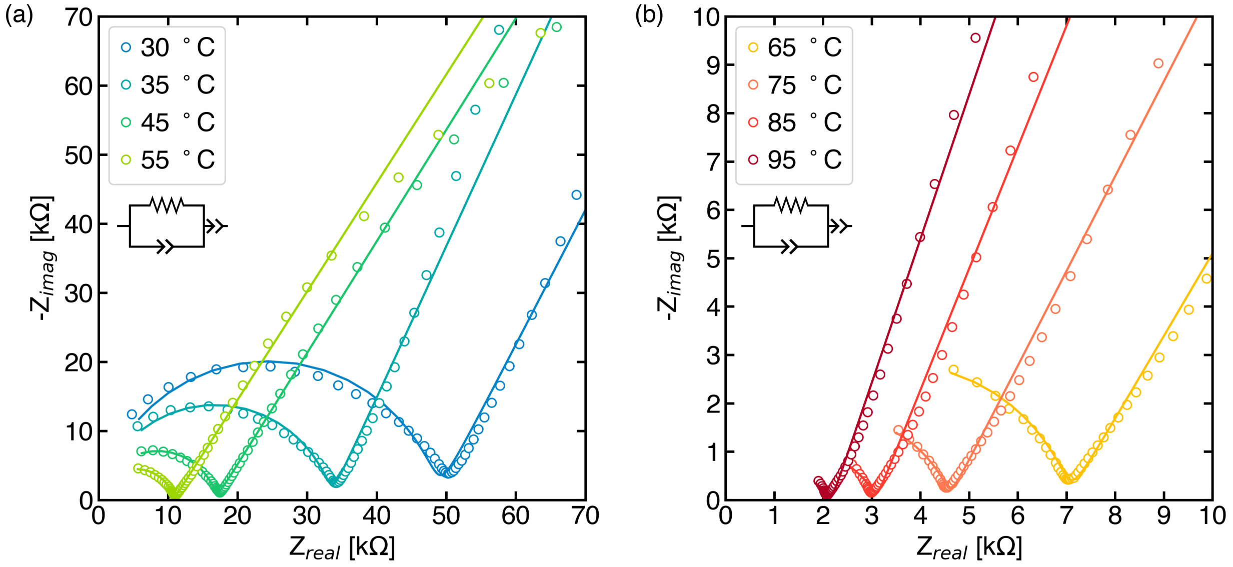

Figure S11: Temperature-dependent Nyquist plots of $Li_{2-2x/3}Zn_{1-x}Zr_{2x/3}Cl_4$ (x = 0.4) from (a) 30 °C to 55 °C and (b) 65 °C to 95 °C fit with an (R_1Q_1) + Q_2 equivalent circuit.

Table S5: Fitted parameters for $Li_{2-2x/3}Zn_{1-x}Zr_{2x/3}Cl_4$ (x = 0.4) of temperature-dependent electrochemical impedance spectroscopy to an $(R_1Q_1) + Q_2$ equivalent circuit, where R_1 is the bulk resistance, Q is a constant phase element, n is the ideality parameter, C is the capacitance, and σ is ionic conductivity.

T (°C)	R_1 (Ω)	σ (S cm^{-1})	n_1	Q_1(S/s^n)	C_1 (F)	τ_1 (Hz)	n_2	Q_2(S/s^n)
30	4.86E+04	1.28E-05	0.88	4.26E-11	6.62E-12	4.95E+05	0.7	1.97E-07
35	3.33E+04	1.87E-05	0.88	4.45E-11	6.63E-12	7.21E+05	0.73	1.86E-07
45	1.68E+04	3.7E-05	0.89	4.26E-11	6.78E-12	1.39E+06	0.65	5.45E-07
55	1.08E+04	5.77E-05	0.88	5.36E-11	6.95E-12	2.12E+06	0.64	8.55E-07
65	7.03E+03	8.86E-05	0.85	8.44E-11	7.03E-12	3.22E+06	0.66	8.9E-07
75	4.59E+03	1.36E-04	0.81	1.76E-10	6.77E-12	5.13E+06	0.7	7.96E-07
85	3.1E+03	2.01E-04	0.73	6.37E-10	5.55E-12	9.26E+06	0.76	5.69E-07
95	2.19E+03	2.84E-04	0.6	6.35E-09	3.79E-12	1.92E+07	0.79	4.99E-07

Nyquist Plots and Fitted Parameters for x = 0.6

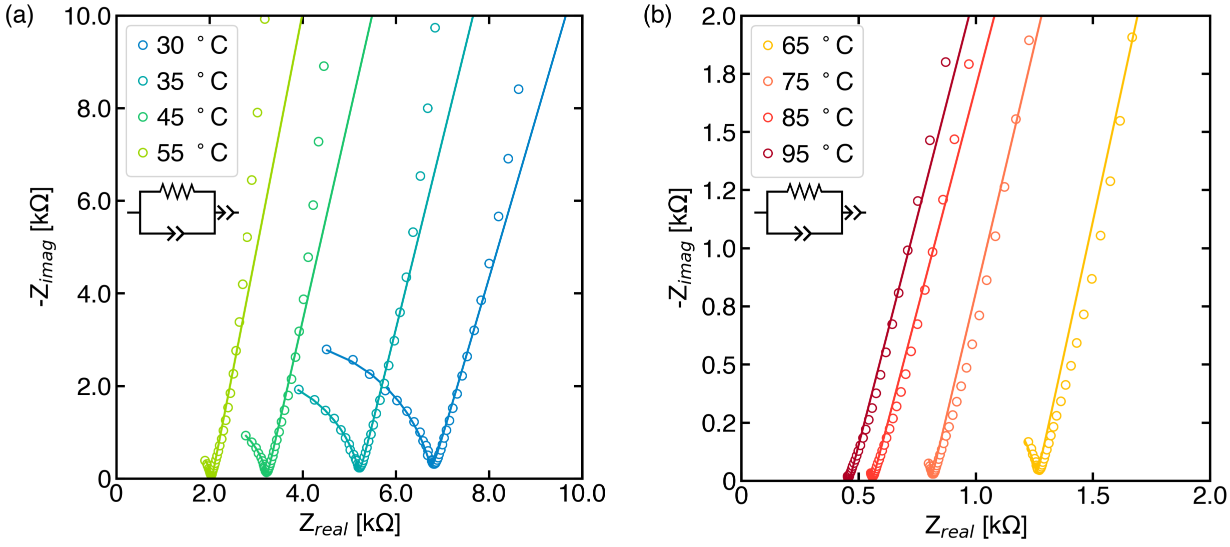

Figure S12: Temperature-dependent Nyquist plots of $Li_{2-2x/3}Zn_{1-x}Zr_{2x/3}Cl_4$ (x = 0.6) from (a) 30 °C to 55 °C and (b) 65 °C to 95 °C fit with an $(R_1Q_1) + Q_2$ equivalent circuit.

Table S6: Fitted parameters for $Li_{2-2x/3}Zn_{1-x}Zr_{2x/3}Cl_4$ (x = 0.6) of temperature-dependent electrochemical impedance spectroscopy to an $(R_1Q_1) + Q_2$ equivalent circuit, where R_1 is the bulk resistance, Q is a constant phase element, n is the ideality parameter, C is the capacitance, and σ is ionic conductivity.

T (°C)	R_1 (Ω)	σ (S cm^{-1})	n_1	Q_1(S/s^n)	C_1 (F)	τ_1 (Hz)	n_2	Q_2(S/s^n)
30	6.74E+03	6.74E-05	0.92	3.04E-11	7.61E-12	3.10E+06	0.82	1.92E-07
35	5.21E+03	8.72E-05	0.91	3.79E-11	7.59E-12	4.02E+06	0.85	1.62E-07
45	3.23E+03	1.41E-04	0.91	3.57E-11	7.76E-12	6.35E+06	0.86	1.67E-07
55	2.05E+03	2.21E-04	0.90	4.15E-11	6.80E-12	1.14E+07	0.88	1.61E-07
65	1.26E+03	3.61E-04	0.90	4.48E-11	7.30E-12	1.73E+07	0.86	2.16E-07
75	8.10E+02	5.61E-04	1	1.09E-11	1.00E-11	1.96E+07	0.85	2.92E-07
85	5.60E+02	8.11E-04	0.95	2.32E-11	9.66E-12	2.94E+07	0.84	4.33E-07
95	4.62E+02	9.85E-04	0.91	3.43E-11	5.98E-12	5.77E+07	0.84	5.11E-07

Nyquist Plots and Fitted Parameters for x = 0.9

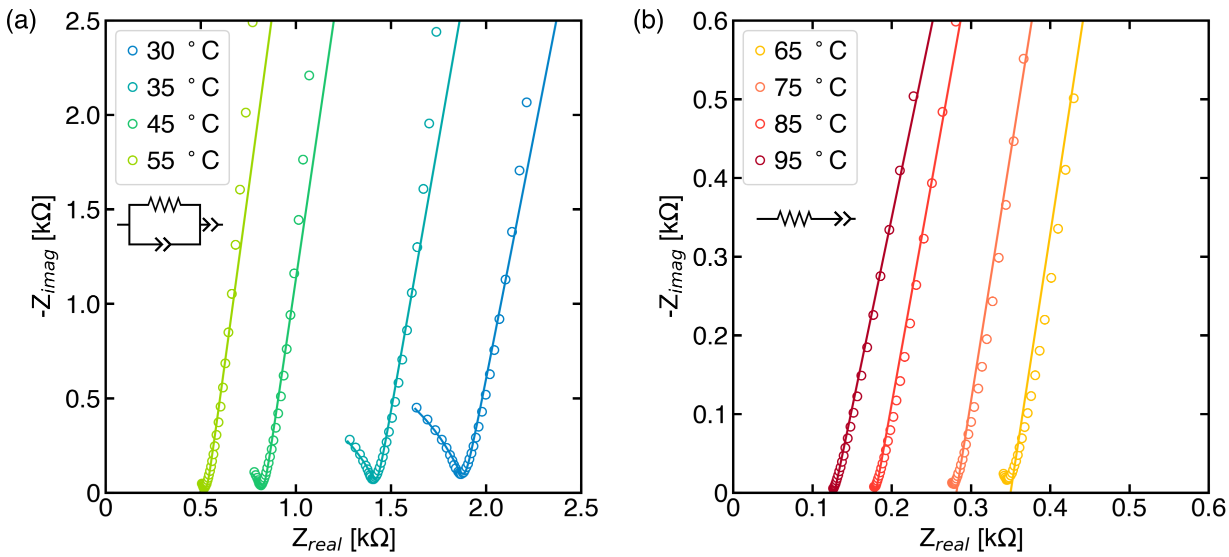

Figure S13: Temperature-dependent Nyquist plots of $Li_{2-2x/3}Zn_{1-x}Zr_{2x/3}Cl_4$ (x = 0.9) from (a) 30 °C to 55 °C with an (R_1Q_1) + Q_2 equivalent circuit and (b) 65 °C to 95 °C fit with an R_1 + Q_2 equivalent circuit.

Table S7: Fitted parameters for $Li_{2-2x/3}Zn_{1-x}Zr_{2x/3}Cl_4$ (x = 0.9) of temperature-dependent electrochemical impedance spectroscopy to $(R_1Q_1)+Q_2$ and R_1 + Q_2 equivalent circuits, where R_1 is the bulk resistance, Q is a constant phase element, n is the ideality parameter, C is the capacitance, and σ is ionic conductivity.

T (°C)	R_1 (Ω)	σ (S cm^{-1})	n_1	Q_1(S/s^n)	C_1 (F)	τ_1 (Hz)	n_2	Q_2(S/s^n)
30	1.89E+03	1.57E-04	0.83	2.07E-10	9.98E-12	8.45E+06	0.89	1.46E-07
35	1.43E+03	2.08E-04	0.79	3.66E-10	9.18E-12	1.21E+07	0.89	1.38E-07
45	8.37E+02	3.55E-04	0.70	2.10E-09	6.42E-12	2.96E+07	0.91	1.31E-07
55	5.36E+02	5.54E-04	0.60	1.17E-08	3.96E-12	7.50E+07	0.91	1.36E-07
65	3.50E+02	8.47E-04	-	-	-	-	0.90	1.67E-07
75	2.82E+02	1.05E-03	-	-	-	-	0.90	1.95E-07
85	1.80E+02	1.65E-03	-	-	-	-	0.89	2.51E-07
95	1.28E+02	2.32E-03	-	-	-	-	0.87	3.53E-07

Nyquist Plots and Fitted Parameters for Li_2ZrCl_6

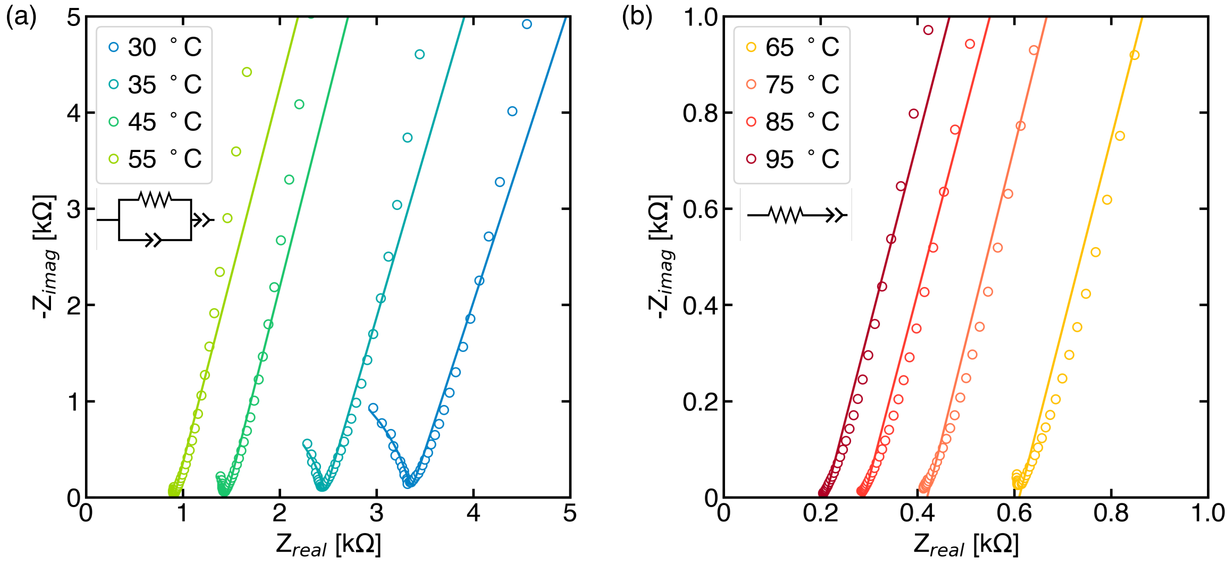

Figure S14: Temperature-dependent Nyquist plots of Li_2ZrCl_6 from (a) 30 °C to 45 °C fit with an (R_1Q_1) + Q_2 and 55 °C fit with an R_1 + Q_2 and (b) 65 °C to 95 °C fit with an R_1 + Q_2 equivalent circuit.

Table S8: Fitted parameters for Li_2ZrCl_6 of temperature-dependent electrochemical impedance spectroscopy to $(R_1Q_1)+Q_2$ and R_1 + Q_2 equivalent circuits, where R_1 is the bulk resistance, Q is a constant phase element, n is the ideality parameter, C is the capacitance, and σ is ionic conductivity.

T (°C)	R_1 (Ω)	σ (S cm^{-1})	n_1	Q_1(S/s^n)	C_1 (F)	τ_1 (Hz)	n_2	Q_2(S/s^n)
30	3.35E+03	1.36E-04	0.91	3.27E-11	6.92E-12	6.87E+06	0.80	3.14E-07
35	2.46E+03	1.85E-04	0.91	3.63E-11	6.93E-12	9.33E+06	0.82	2.91E-07
45	1.45E+03	3.13E-04	0.84	1.22E-10	6.49E-12	1.69E+07	0.84	2.75E-07
55	9.10E+02	4.99E-04	-	-	-	-	0.84	3.28E-07
65	6.10E+02	7.45E-04	-	-	-	-	0.84	3.74E-07
75	4.20E+02	1.08E-03	-	-	-	-	0.85	4.26E-07
85	2.90E+02	1.57E-03	-	-	-	-	0.84	5.56E-07
95	2.10E+02	2.16E-03	-	-	-	-	0.84	6.46E-07

Fitting EIS Data with an $(R_1Q_1) + (R_2Q_2) + Q_3$ Equivalent Circuit

As shown in Figure S15, at low frequencies, the capacitive tail changes slope, resulting in poor fitting of the constant phase element Q_2. An additional semicircle feature (R_2Q_2) may help improve the overall fit of the impedance. In a system with an ideal capacitor and perfect interfacial contact between the electrode and electrolyte, two distinct semicircles are present: the first being a result of bulk ion transport and the second being representative of grain boundary contributions. However, in many cases, these semicircles are depressed, making it harder to distinguish between the two.[5] Given the poorly-defined nature of the second semicircle feature in these Li-Zn-Zr-Cl materials, we opted to use a more straightforward equivalent circuit ($(R_1Q_1) + Q_2$) to minimize risks of over-fitting the data.

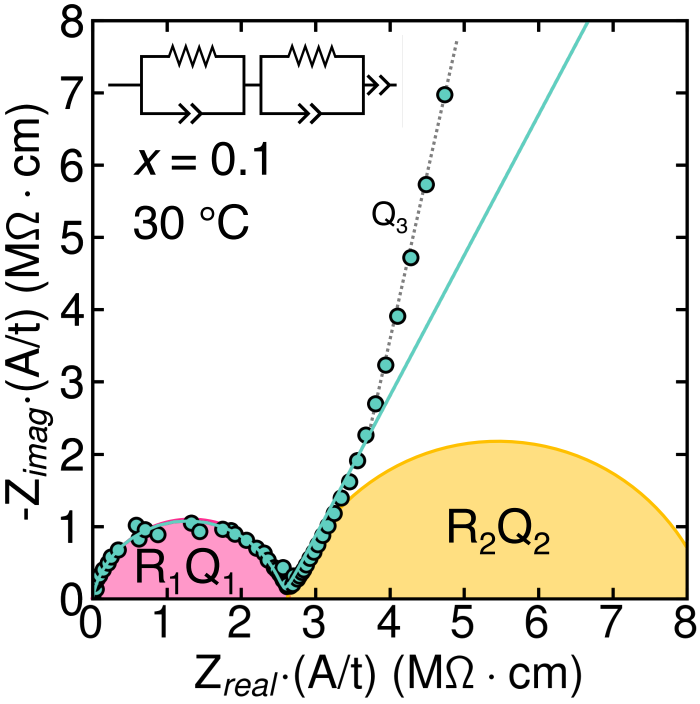

Figure S15: Representative example of an (R_1Q_1) + (R_2Q_2) + Q_3 equivalent circuit fit on Nyquist plot at 30 °C for the composition at $x = 0.1$. The solid light blue line represents the original fit using an (R_1Q_1) + Q_2 equivalent circuit, where at low frequencies, Q_2 does not adequately fit the capacitive tail. The dotted gray line depicts the expected fit for the capacitive tail with an additional (RQ) element implemented in the equivalent circuit.

Structural Refinements

Monoclinic $P2_1/c$ Fit of $Li_{2-2x/3}Zn_{1-x}Zr_{2x/3}Cl_4$ at x = 0.3

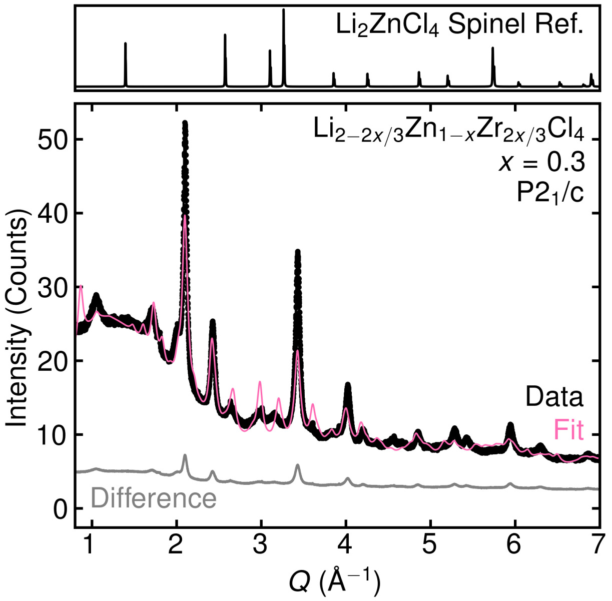

Figure S16: Rietveld refinement of monoclinic distorted spinel (space group $P2_1/c$) with synchrotron X-ray diffraction for $Li_{2-2x/3}Zn_{1-x}Zr_{2x/3}Cl_4$ ($x = 0.3$).

Figure S16 shows $Li_{2-2x/3}Zn_{1-x}Zr_{2x/3}Cl_4$ at x = 0.3 fit with a monoclinic $P2_1/c$ space group. Similar to Artal *et al.*, all Zr^{4+} cations share octahedral sites with Li^+ while Zn^{2+} remained in tetrahedral positions.[6] Structural distortion from the cubic spinel structure results in poor modeling of the SXRD data, particularly at high Q. The cubic $Fd\overline{3}mZ$ spinel structure more accurately describes the SXRD data.

Vesta Models for $Li_{2-2x/3}Zn_{1-x}Zr_{2x/3}Cl_4$ (x = 0.0, 0.1, 0.3, 0.6)

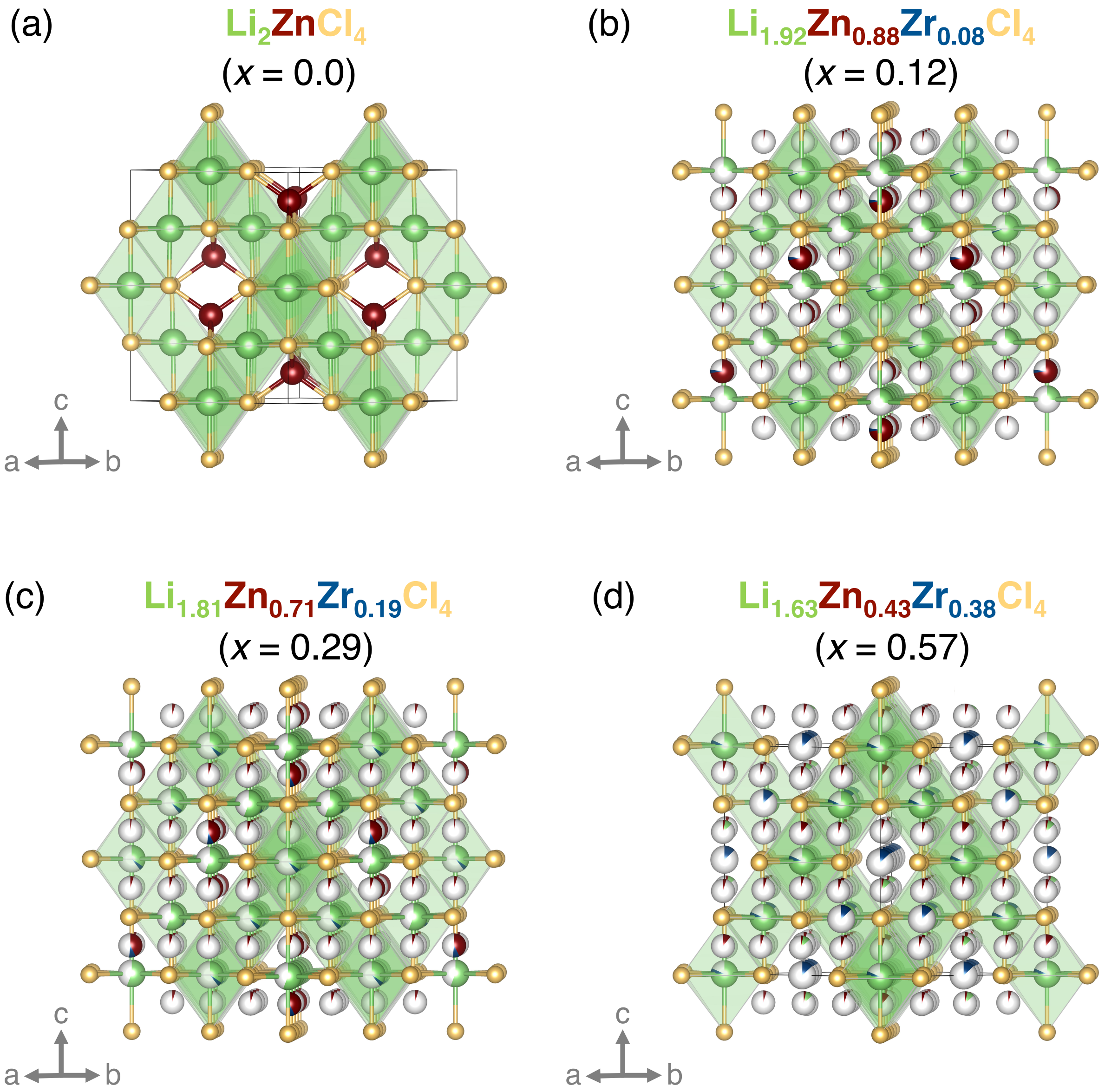

Figure S17: Structural models of $Li_{2-2x/3}Zn_{1-x}Zr_{2x/3}Cl_4$ at (a) $x = 0$, (b) $x = 0.1$, (c) $x = 0.3$, and (d) $x = 0.6$ from joint refinements against neutron pair distribution analysis and synchrotron powder X-ray diffraction data. Li^+ atoms are green, Zn^{2+} are red, Zr^{4+} are blue, and Cl^- atoms are yellow.

Joint Refinement Parameters

To maintain stoichiometry in compositions throughout joint refinements, all cation occupancies are constrained based on Zr^{4+} concentration across the series $Li_{2-2x/3}Zn_{1-x}Zr_{2x/3}Cl_4$. A more detailed example of the exact refinement procedure is demonstrated below with the composition at $x = 0.3$. To start, a range is assigned to the overall Zr^{4+} concentration. We elected to use a range of $x = 0.3 \pm 0.5$ as this proved to be a wide enough parameter space for occupancies to reside within the limits without getting stuck at the edges. As Zr^{4+} content is equal to $2x/3$, this produces a concentration range of 0.17-0.23. The stated range acts as the minimum and maximum value constraints set to Zr^{4+} occupancy on the 16*d* sites relative to Zr^{4+} concentration in other Wyckoff positions. The remaining sites all have similar maximum concentration equations as the 16*d* site, while minimum values are set to 0 to ensure refinements do not result in negative site occupancies.

Table S9: Minimum and maximum constraints used for Zr4+ joint nPDF/SXRD refinements of fractional occupancy in the 16*d*, 16*c*, 8*a*, 48*f*, and 8*b* sites. Equations are written to account for differences in multiplicity across the different Wyckoff positions.

Cation Site	min	max
Zr_{16d}	$(0.17/2)$-Zr_{16c}-$(Zr_{8a}/2)$-$Zr_{48f}(3)$-$(Zr_{8b}/2)$	$(0.23/2)$-Zr_{16c}-$(Zr_{8a}/2)$-$Zr_{48f}(3)$-$(Zr_{8b}/2)$
Zr_{16c}	0	$(0.23/2)$-Zr_{16d}-$(Zr_{8a}/2)$-$Zr_{48f}(3)$-$(Zr_{8b}/2)$
Zr_{8a}	0	0.23-$Zr_{16c}(2)$-$Zr_{16c}(2)$-$Zr_{48f}(6)$-Zr_{8b}
Zr_{48f}	0	$(0.23/6)$-$(Zr_{16c}/3)$-$(Zr_{16c}/3)$-$(Zr_{8a}/6)$-$(Zr_{8b}/6)$
Zr_{8b}	0	0.23-$Zr_{16c}(2)$-$Zr_{16c}(2)$-$Zr_{48f}(6)$-Zr_{8a}

Li^+ occupancy follows the equation 2-2*x*/3, or 2 minus the total concentration of Zr^{4+}. From this, Li^+ occupancy in the 16*d* site can be described with Equation 1. The Li_{16d} occupancy is fixed to Equation 1 while the remaining Li^+ sites, including Li_{16c}, Li_{8a}, Li_{8b}, and Li_{48f} are allowed to freely refine given minimum and maximum values of 0 and 1, respectively.

$$Li_{16d} = \left[\frac{2-(2Zr_{16d}+2Zr_{16c}+Zr_{8a}+Zr_{8b}+6Zr_{48f}}{2}\right] - Li_{16c} - \frac{Li_{8a}}{2} - \frac{Li_{8b}}{2} - 3Li_{48f} \qquad (1)$$

Similar to Li^+, 8*a* Zn^{2+} is fixed to an equation in terms of Zr^{4+} occupancy. Given that

x=3[Zr^{4+}]/2 and Zn^{2+}=1-x, we can describe Zn^{2+} occupancy using Equation 2. Other available Zn^{2+} sites including Zn_{16d}, Zn_{16c}, Zn_{8b}, and Zn_{48f} are only constrained to a fractional occupancy range of 0 to 1.

$$Zn_{8a} = \left(1 - \left[\frac{3(2Zr_{16d} + 2Zr_{16c} + Zr_{8a} + Zr_{8b} + 6Zr_{48f}}{2}\right]\right) - Zn_{8b} - 2Zn_{16d} - 2Zn_{16c} - 6Zn_{48f} \tag{2}$$

We recognize refinement constraints for the remaining Li^{+} and Zn^{2+} sites could be more strict, such as setting maximum values relative to total Zr^{4+} content. However, we opted for less restrictive parameters to minimize bias. Furthermore, the refined occupancies in unconstrained sites did not exceed the allowed total fractional occupancies of the cation, resulting in reasonable values without intense restrictions.

Li_2ZnCl_4 Refined Parameters

Table S10: Results of joint nPDF and SXRD refinement for Li_2ZnCl_4 shown in Figure S17a. Space group $Fd\bar{3}mZ$, $a = 10.34352(3)$ Å. nPDF $R_{wp} = 7.99\%$, SXRD $R_{wp} = 5.98\%$.

Site	Atom	*x*	*y*	*z*	Occupancy	Beq
16*d*	Li	0.5	0.5	0.5	1.00	1.12(1)
8*a*	Zn	0.125	0.125	0.125	1.00	2.28(2)
32*e*	Cl	0.25245(4)	0.25245(4)	0.25245(4)	1.00	1.326(4)

$Li_{1.92}Zn_{0.88}Zr_{0.08}Cl_4$ (x = 0.12) Refined Parameters

Table S11: Results of dual nPDF and SXRD refinement for $Li_{1.92}Zn_{0.88}Zr_{0.08}Cl_4$ ($x = 0.12$) shown in Figure S17b. Space group $Fd\bar{3}mZ$, $a = 10.3478(1)$ Å. nPDF $R_{wp} = 9.05\%$, SXRD $R_{wp} = 3.99\%$.

Site	Atom	*x*	*y*	*z*	Occupancy	Beq
16*d*	Li	0.5	0.5	0.5	0.630(1)	1.568(1)
16*d*	Zr	0.5	0.5	0.5	0.0169(7)	1.568(1)
16*c*	Li	0.0	0.0	0.0	0.27(1)	4.2(2)
8*a*	Zn	0.125	0.125	0.125	0.7267(6)	2.2561(3)
8*a*	Zr	0.125	0.125	0.125	0.046(4)	2.2561(3)
8*b*	Zn	0.375	0.375	0.375	0.0139(9)	1.0(1)
48*f*	Zn	0.125	0.125	0.875	0.0232(5)	1.0(1)
32*e*	Cl	0.25285(5)	0.25285(5)	0.25285(5)	1.00	1.65(1)

$Li_{1.81}Zn_{0.71}Zr_{0.19}Cl_4$ (x = 0.29) Refined Parameters

Table S12: Results of dual nPDF and SXRD refinement for $Li_{1.81}Zn_{0.71}Zr_{0.19}Cl_4$ ($x = 0.29$) shown in Figure S17c. Space group $Fd\bar{3}mZ$, $a = 10.3570(2)$ Å. nPDF $R_{wp} = 11.87\%$, SXRD $R_{wp} = 2.76\%$.

Site	Atom	*x*	*y*	*z*	Occupancy	Beq
16*d*	Li	0.5	0.5	0.5	0.344(1)	1.263(1)
16*d*	Zr	0.5	0.5	0.5	0.0516(5)	1.263(1)
16*c*	Li	0.0	0.0	0.0	0.56(2)	3.18(8)
8*a*	Zn	0.125	0.125	0.125	0.4240(3)	2.9186(3)
8*a*	Zr	0.125	0.125	0.125	0.091(5)	2.9186(3)
8*b*	Zn	0.375	0.375	0.375	0.021(1)	4.9(8)
48*f*	Zn	0.125	0.125	0.875	0.0438(3)	3.4(2)
32*e*	Cl	0.25212(7)	0.25212(7)	0.25212(7)	1.00	2.04(2)

$Li_{1.63}Zn_{0.43}Zr_{0.38}Cl_4$ (x = 0.57) Refined Parameters

Table S13: Results of dual nPDF and SXRD refinement for $Li_{1.63}Zn_{0.43}Zr_{0.38}Cl_4$ ($x = 0.57$) shown in Figure S17d. Space group $Fd\bar{3}mZ$, $a = 10.4071(2)$ Å. nPDF $R_{wp} = 12.92\%$, SXRD $R_{wp} = 2.41\%$.

Site	Atom	*x*	*y*	*z*	Occupancy	Beq
16*d*	Zr	0.5	0.5	0.5	0.130(2)	1.90(7)
16*c*	Li	0.0	0.0	0.0	0.76(1)	4.8215(8)
16*c*	Zr	0.0	0.0	0.0	0.059(2)	4.8215(8)
8*a*	Zn	0.125	0.125	0.125	0.11(4)	5.0(4)
8*b*	Zn	0.375	0.375	0.375	0.034(3)	3.60(9)
8*b*	Li	0.375	0.375	0.375	0.10(1)	3.60(9)
48*f*	Zn	0.125	0.125	0.875	0.047(2)	4.5(1)
32*e*	Cl	0.2538(1)	0.2538(1)	0.2538(1)	1.00	2.75(3)

POWGEN Neutron Powder Diffraction

Neutron powder diffraction (NPD) data were collected on the POWGEN diffractometer at the Spallation Neutron Source, Oak Ridge National Laboratory, as shown in Figure S18. The NPD data are well-described by the disordered structures determined from joint refinements of the SXRD/nPDF data. We note that the POWGEN data were collected on different sample batches from the SXRD/nPDF data; as such, we observe slight deviations in the lattice parameters. Rietveld refinements shown in Figure S18 were performed by fixing the occupancies of the cations to the disordered models and allowing the lattice parameters to refine.

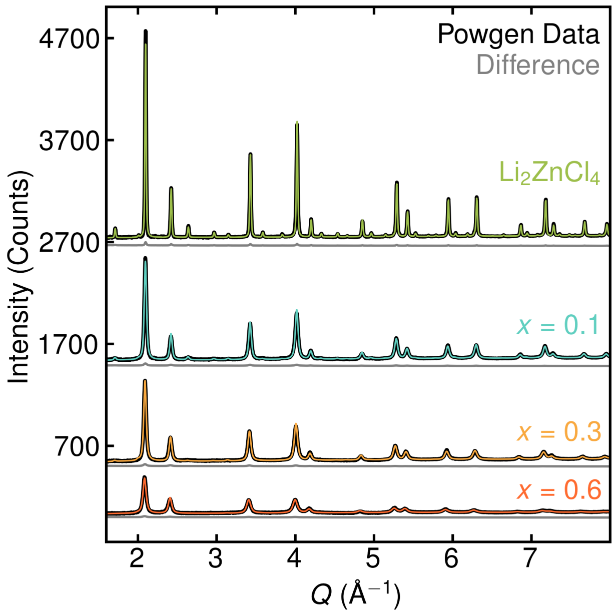

Figure S18: Rietveld refinements of neutron powder diffraction (NPD) data collected on the POWGEN diffractometer at SNS ORNL. Data are shown in black, fits are colored lines, and the difference curve is shown in gray.

Table S14: Refined lattice parameters and R_{wp} values from POWGEN Rietveld refinements for $Li_{2-2x/3}Zn_{1-x}Zr_{2x/3}Cl_4$ for x = 0.0, 0.1, 0.3, 0.6

Composition	Refined x	Lattice Parameter (Å)	R_{wp}
Li_2ZnCl_4	0	10.34352(3)	3.65%
$Li_{1.92}Zn_{0.88}Zr_{0.08}Cl_4$	0.12	10.3555(2)	3.24%
$Li_{1.81}Zn_{0.71}Zr_{0.19}Cl_4$	0.29	10.3801(2)	3.14%
$Li_{1.63}Zn_{0.43}Zr_{0.38}Cl_4$	0.57	10.4076(2)	2.62%

x = 0.6 Joint Refinement Goodness of Fit by 16*d* Li^+ Concentration

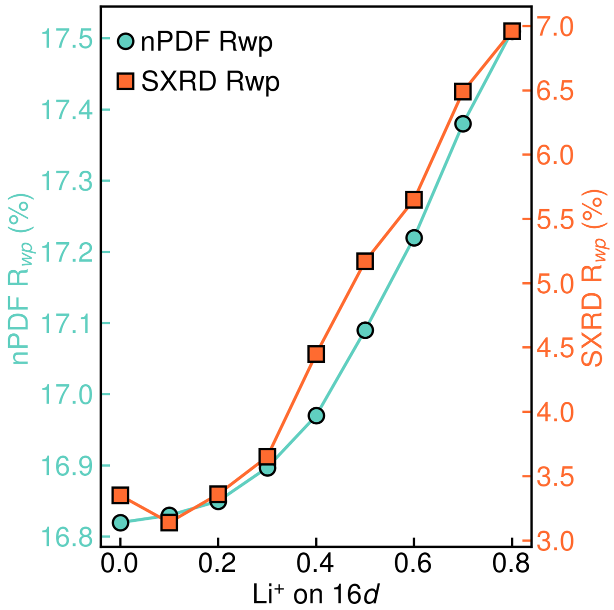

Figure S19: R_{wp} values extracted from weighted joint nPDF/SXRD refinement as a function of Li^+ concentration on the 16*d* site. Li^+ occupancy on the 16*d* site was fixed while other cations were allowed to freely refine under the conditions explained in Table S9.

x = 0.3 SXRD R_{wp} Heat Maps

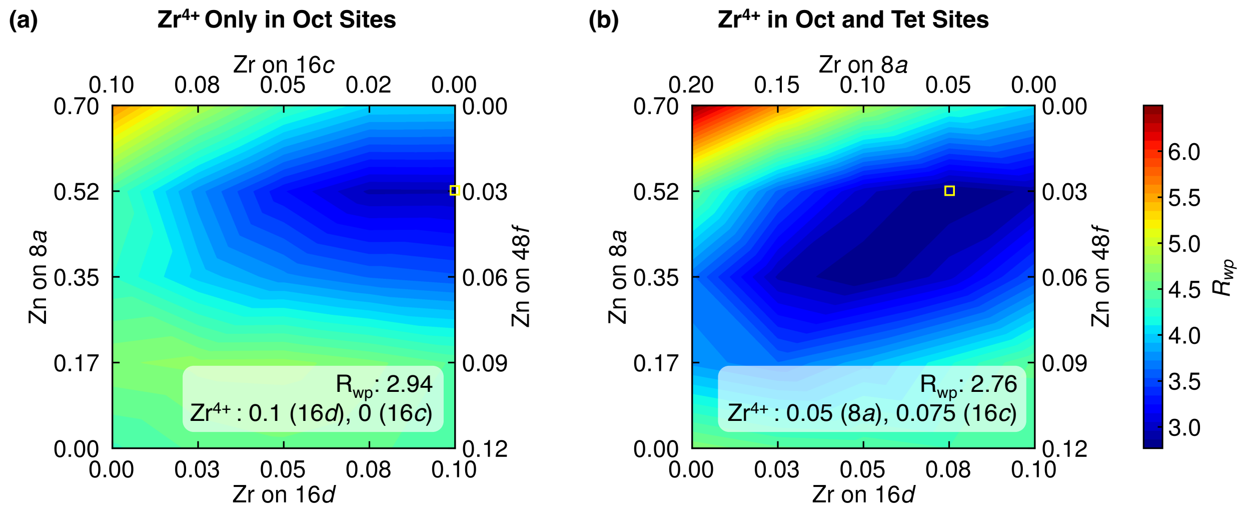

Figure S20: R_{wp} values extracted from SXRD refinements with select Zn^{2+} 8*a*/48*f* occupancies and (a) Zr^{4+} on only octahedral sites (16*d*/16*c*) and (b) Zr^{4+} on octahedral and tetrahedral sites (16*d*/8*a*). Lowest R_{wp} values in each plot are represented with an open yellow box.

To lend support to our determination of the cation disorder, we determined the R_{wp} from the SXRD data for structural models in which Zr^{4+} occupies octahedral sites (Figure S20a) and both octahedral and tetrahedral sites (Figure S20b) for the $x = 0.3$ composition. Zr^{4+} occupancies were allowed to vary systematically against various Zn^{2+} 8*a*/48*f* occupancies, and the R_{wp} values for each combination of cation occupancies are shown as a color map. Li^+ was fixed to occupancies of 0.344 and 0.557 in the 16*d* and 16*c* sites, respectively. The lattice parameter was fixed to the value determined by Pawley fit, while all other parameters were allowed to freely refine. The total occupancies of Zn^{2+} and Zr^{4+} were constrained to the series formula $Li_{2-2x/3}Zn_{1-x}Zr_{2x/3}Cl_4$ for $x = 0.3$. Figure S20a illustrates the best fit (lowest R_{wp}) occurs when no Zr^{4+} resides in the 16*c* site and Zn^{2+} occupies both the 8*a* and 48*f* positions. In contrast, Figure S20b shows a wider range of low R_{wp} values ($< 3.5\%$). This suggests that Zr^{4+} residing in the 8*a* and 16*d* sites provides an improved description of the SXRD data compared to a model in which Zr^{4+} resides only octahedral sites. We note that this analysis only takes into account the SXRD data and does not consider the local structure information from nPDF. Although there are structural models with slightly different configurations of cation disorder that achieve similar fit statistics, we are confident that our refined

models across the series correspond to the global minimum in the fits.

Raman Spectroscopy

Measurement Details for Raman Spectroscopy

Table S15: Raman spectroscopy instrumental parameters. Laser intensity changed per sample to increase Raman signal strength while minimizing risk of instrument damage. Aperture and resolution parameters remain constant across all samples.

Composition	x	Intensity (W)	Aperture	Resolution (cm^{-1})
Li_2ZnCl_4	0.00	0.05	37	2
$Li_{1.92}Zn_{0.88}Zr_{0.08}Cl_4$	0.12	0.30	37	2
$Li_{1.81}Zn_{0.71}Zr_{0.19}Cl_4$	0.29	0.15	37	2
$Li_{1.63}Zn_{0.43}Zr_{0.38}Cl_4$	0.57	0.10	37	2
$Li_{1.40}Zn_{0.10}Zr_{0.60}Cl_4$	0.90	0.05	37	2
Li_2ZrCl_6	1.00	0.15	37	2

Bond Valence Sums

Bond Valence Sum Calculation Information

Table S16: Bond Valence Sum Calculation Information including the bond valence sums for each crystallographic site and averaged values over matching coordination environments. Constants used were b = 0.37, Li R_0 = 1.91, Zn R_0 = 2.01, Zr R_0 = 2.33.[7]

x	Cation	16d V_i	16c V_i	8a V_i	48f V_i	8b V_i	Tet Ave. BVS	Oct Ave. BVS
0.0	Li^+	1.03	0.90	1.46	1.64	1.86	1.65	0.97
0.0	Zn^{2+}	1.35	1.18	1.91	2.15	2.44	2.17	1.27
0.0	Zr^{4+}	3.22	2.80	4.54	5.11	5.80	5.15	3.01
0.1	Li^+	1.04	0.89	1.43	1.43	1.88	1.65	0.97
0.1	Zn^{2+}	1.37	1.16	1.87	2.15	2.46	2.16	1.26
0.1	Zr^{4+}	3.24	2.76	4.44	5.10	5.85	5.13	3.00
0.3	Li^+	1.01	0.90	1.47	1.63	1.81	1.63	0.96
0.3	Zn^{2+}	1.33	1.18	1.93	2.13	2.37	2.14	1.26
0.3	Zr^{4+}	3.16	2.80	4.57	5.07	5.62	5.09	2.98
0.6	Li^+	0.96	0.89	1.48	1.58	1.69	1.58	0.93
0.6	Zn^{2+}	1.26	1.16	1.94	2.07	2.23	2.08	1.21
0.6	Zr^{4+}	2.99	2.77	4.60	4.92	5.29	4.93	2.88

Bond valence sums (V_i) act as a simple metric to probe preferred coordination environments using the equation $V_i = \sum_j s_{ij}$, where $s_{ij} = \exp\left(\frac{R_0 - R_{ij}}{b}\right)$. In this calculation, R_0 and b are constants, while R_{ij} describes the bond length between i and j. To enable an adequate visualization of preferred environments, all cations across the substitution series are subjected to BVS analysis in both octahedral and tetrahedral environments regardless of the jointly refined occupancies. Cation-Cl^- distances were calculated based on the relative site positions and lattice parameters of the disordered model structures. Given the specific coordinates of Cl^- create variance in bond distances between Wyckoff positions of equivalent coordination, bond valence sums are averaged among all tetrahedral and octahedral sites for a more holistic representation of site preference (Table S16).

References

(1) Kuske, P.; Schäfer, W.; Lutz, H. Neutron diffraction studies on spinel type Li_2ZnCl_4. *Mater. Res. Bull.* **1988**, *23*, 1805–1808.

(2) Solinas, I.; Lutz, H. Nonceramic Preparation Techniques for Ternary Halides AB_2X_4 with A = Mg, Mn, Zn; B = Li, Na; X = Cl, Br. *J. Solid State Chem.* **1995**, *117*, 34–38.

(3) Rom, C. L.; Yox, P.; Cardoza, A. M.; Smaha, R. W.; Phan, M. Q.; Martin, T. R.; Maughan, A. E. Expanding the Phase Space for Halide-Based Solid Electrolytes: Li–Mg–Zr–Cl Spinels. *Chem. Mater.* **2024**, *36*, 7283–7291.

(4) Meyer, W.; Neldel, H. Über die Beziehungen. *Z. Techn. Phys.* **1937**, *18*, 588–93.

(5) Braun, P.; Uhlmann, C.; Weber, A.; Störmer, H.; Gerthsen, D.; Ivers-Tiffée, E. Separation of the bulk and grain boundary contributions to the total conductivity of solid lithium-ion conducting electrolytes. *Journal of Electroceramics* **2017**, *38*, 157–167.

(6) Artal, R.; Andersen, H. L.; Del Olmo, R.; Villaluenga, I.; Sobrados, I.; Diez-Gómez, V.; Gainza, J.; Fernández-Diaz, M. T.; Alonso, J. A.; Jimenez, R.; Aguadero, A. Cation disorder and lithium conductivity in mechanochemically synthesized chloride solid electrolytes. *Solid State Ionics* **2025**, *428*, 116952.

(7) Brese, N.; O'keeffe, M. Bond-valence parameters for solids. *Struct. Sci.* **1991**, *47*, 192–197.
